# Chalcogenide Materials in Water Purification: Advances in Adsorptive and Photocatalytic Removal of Organic Pollutants

**DOI:** 10.1002/smll.202501378

**Published:** 2025-05-28

**Authors:** Damilola Caleb Akintayo, Tunde Lewis Yusuf, Nonhlangabezo Mabuba

**Affiliations:** ^1^ Department of Chemical Sciences Faculty of Science University of Johannesburg, Doornfontein Campus Johannesburg 2028 South Africa; ^2^ Department of Chemistry Faculty of Natural and Agricultural Sciences University of Pretoria Private Bag X20 Hatfield Pretoria 0028 South Africa; ^3^ Center for Nanomaterials Science Research Faculty of Science University of Johannesburg, Doornfontein Campus Johannesburg 2028 South Africa

**Keywords:** adsorption, metal chalcogenides, organic pollutants, photocatalytic, water/wastewater treatment

## Abstract

Chalcogenide‐based materials, known for their unique physicochemical properties, emerge as promising solutions for the removal of hazardous organic pollutants, such as dyes, pharmaceuticals, pesticides, and herbicides, from water and wastewater. This review examines the latest developments in the synthesis, structural optimization, and application of chalcogenide materials for environmental remediation. The past decade has witnessed remarkable advances in controlling the composition and structure of chalcogenide materials at the atomic level. The development of precise synthetic methods enables the creation of complex hierarchical structures, heterojunctions, and hybrid materials, leading to significant improvements in photocatalytic efficiency, stability, and selectivity for various environmental applications. Key emphasis is placed on adsorption and photocatalysis as green technologies, offering efficient pathways for pollutant removal. Mechanistic insights into the interactions between chalcogenide materials and contaminants are explored, providing a comprehensive understanding of their performance. Furthermore, challenges such as toxicity, scalability, and operational stability are discussed alongside future prospects for integrating these materials into industrial‐scale water treatment systems. This review aims to inspire continued innovation in sustainable water purification technologies using chalcogenides.

## Graphical abstract

Chalcogenide materials emerge as efficient agents for water purification, enabling adsorptive and photocatalytic removal of dyes, pharmaceuticals, and pesticides. This review highlights recent advances in synthesis, structural tuning, and pollutant interaction mechanisms, while addressing challenges of toxicity and scalability. Insights into the future integration of chalcogenides in sustainable water treatment technologies are also provided.

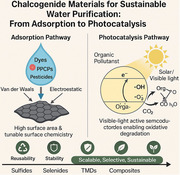

## 1 Introduction

Industrialization is a cornerstone of economic development and societal progress, serving as a fundamental requirement across all sectors and a key determinant of economic standing. However, the rapid expansion of industrial activities has become a major contributor to the release of unprecedented pollutants into the ecosystem, leading to significant environmental consequences across all major habitats.^[^ ^1^, ^3^ ^]^ Water pollution, responsible for an estimated 2.2 million deaths annually,^[^ ^4^ ^]^ has become a critical global issue, endangering human health, aquatic ecosystems, and biodiversity.^[^ ^5^, ^6^, ^7^, ^8^, ^9^ ^]^ Industrial effluents, agricultural runoff and household sewage have contributed a wide range of pollutants,^[^ ^10^, ^11^, ^12^ ^]^ including dyes, pesticides, pharmaceuticals, and personal care products (PPCPs) and unidentified organic substances that are degrading water quality,^[^ ^13^, ^14^, ^15^ ^]^ many of which resist conventional wastewater treatment.^[^ ^16^, ^17^, ^18^ ^]^

Beyond the immediate health risks, the presence of persistent organic pollutants in water bodies destabilizes aquatic ecosystems and worsens water scarcity.^[^ ^19^ ^]^ Traditional water and wastewater treatment methods, including adsorption, chemical oxidation, electrochemical processes, and advanced filtration strategies, have shown limited efficacy in degrading persistent organic contaminants.^[^ ^20^, ^21^, ^22^ ^]^ In addition to traditional methods, microorganisms have been increasingly utilized in water remediation.^[^ ^23^ ^]^ Microbial water treatment involves various enzymatically mediated processes, including metal precipitation and enzymatic ligand synthesis, as well as reduction and oxidation reactions.^[^ ^24^ ^]^ These challenges necessitate the exploration of innovative materials and methods to address water pollution effectively.

In recent years, there has been growing interest in the potential of nanomaterials, characterized by dimensions typically smaller than 100 nm, to transform and enhance water purification and treatment technologies.^[^ ^25^, ^26^, ^27^ ^]^ Carbon and oxide‐based materials such as activated carbon, metal oxides, and mesoporous structures possessing unique physical and chemical properties well‐suited to enhance pollutant removal have been the focus of mainstream study.^[^ ^28^, ^29^, ^30^, ^31^, ^32^, ^33^, ^34^ ^]^ However, their chemical flexibility is often limited, which hinders improved control over catalytic reactivity, surface functionality, and bandgap properties. To address these drawbacks, advanced materials such as metal chalcogenides, carbides, nitrides, covalent–organic frameworks, and hybridized metal–organic frameworks have been developed.^[^ ^35^, ^36^, ^37^, ^38^ ^]^ These porous materials offer exceptional physicochemical properties and hold promise for multifunctional applications.^[^ ^39^, ^40^, ^41^, ^42^ ^]^Additionally, materials like zerovalent iron particles, carbonaceous substances, noble metal nanoparticles, metal oxides, quantum dots, and nanocomposites are widely used in photocatalysis, Fenton‐like processes, membrane filtration, and adsorption.^[^ ^43^, ^44^ ^]^

Chalcogenides, derived from Group XVI elements^[^ ^45^ ^]^ (oxygen, sulfur, selenium, tellurium, and polonium),^[^ ^46^ ^]^ are primarily associated with sulfides, selenides, tellurides, and polonides rather than oxides due to their distinct chemical properties.^[^ ^14^, ^47^, ^48^ ^]^ Chalcogenide materials, known for their stability, affordability, accessibility, and wide bandgaps, are categorized as transition metal oxides (TMOs).^[^ ^48^ ^]^ In contrast, less stable chalcogenides with narrower bandgaps, such as M‐S, M‐Te, and M‐Se, are classified as transition metal chalcogenides (TMCs).^[^ ^49^ ^]^ TMC and its composites have been further classified into four main categories: binary (such as CuS, CdS, ZnTe, and ZnSe), ternary (CuFeS_2_ and Cu_2_WS_4_), quaternary (Cu_2_ZnSnSe_4_ and Cu_2_FeSnS_4_), and heterostructures (AgInS_2_/SnIn_4_S).^[^ ^49^ ^]^ Each category may be loaded (doped) with a conductor to improve functionality. These materials have garnered significant research interest for their remarkable optical absorption, magnetic properties, catalytic activity, and electron mobility.^[^ ^50^, ^51^ ^]^ Their versatile elemental compositions, adjustable bandgaps, abundance in nature, favorable optoelectrical characteristics, catalytic stability and activity in visible light have made them highly suitable for addressing complex water contamination issues through adsorption and photocatalysis.^[^ ^52^ ^]^ Chalcogenide materials, such as CuS, ZnSe, and CdS, have demonstrated significant potential in degrading dyes, PPCPs, pesticides, and other emerging organic pollutants.^[^ ^53^, ^54^ ^]^

The synthesis of TMCs has advanced significantly, employing techniques such as hydrothermal and solvothermal methods, facile sol‐assisted dip‐coating, microwave‐assisted synthesis, sol–gel, solvent‐mixing, cation exchange and electrospinning methods.^[^ ^45^, ^55^, ^56^, ^57^, ^58^, ^59^ ^]^ These methods enable precise control over the size, morphology, and surface properties of the materials, enhancing their performance in water treatment applications. Additionally, the development of composites and heterostructures incorporating chalcogenides, such as ternary and quaternary systems, further expands their functionality and efficiency.

This review aims to provide a comprehensive overview of the applications of chalcogenide materials in water and wastewater treatment, focusing on their roles in adsorption and photocatalytic degradation of organic pollutants. By highlighting recent advancements in the synthesis and utilization of chalcogenides, this review underscores their potential as innovative alternatives to conventional treatment methods. Furthermore, it delves into the mechanisms underlying their interactions with contaminants, offering insights into their efficiencies and multifunctional capabilities. By addressing the ongoing challenges posed by persistent organic pollutants, this review seeks to contribute to the development of sustainable and effective water remediation strategies. This review adopts a systematic approach by conducting a literature search using keywords such as “dyes,” “pharmaceutical and personal care products,” “pesticides,” “chalcogenides,” “adsorption,” and “photodegradation.” Inclusion criteria focused on quantitative studies published in peer‐reviewed journals, employing primary data, and addressing adsorption and photocatalytic degradation of dyes, pharmaceuticals, personal care products, and pesticides. Only articles written in English were considered. Studies were excluded if they were qualitative, published in languages other than English, or unrelated to the adsorption and photodegradation of pollutants using chalcogenide materials.

## 2 Evolution, Properties, and Applications of Metal Chalcogenides

### 2.1 Evolution of Metal Chalcogenides

**Figure 1:**
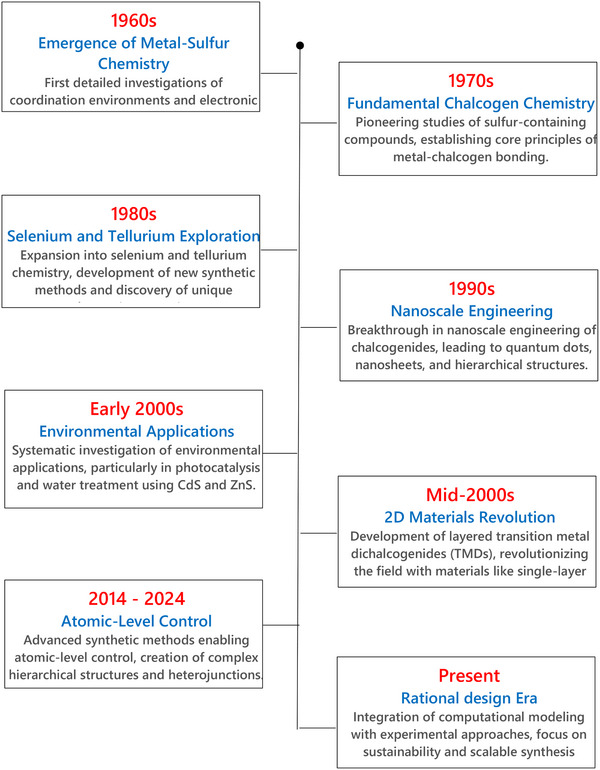
Evolution of Metal Chalcogenides Research.

The evolution of chalcogenide materials in environmental applications represents a fascinating journey from fundamental chemistry to advanced functional materials (**Figure** **1**). Their chemistry began in the early 1970s^[^ ^60^, ^61^, ^62^ ^]^ with pioneering investigation documented on sulfur‐containing compounds due to its abundance and relative stability, establishing fundamental principles of metal–chalcogen bonding.^[^ ^63^ ^]^ As shown in Figure 1, the early application (pre‐2000s) was focused on the initial investigation centered on metal sulfide chalcogenides, particularly MoS₂ and ZnS, with an emphasis on their electronic and photoconductive characteristics while the corresponding environmental application was primarily confined to fundamental water purification methods employing ZnS for removing of heavy metal ions and photocatalytic processes under UV illumination.

Systematic investigations into the environmental applications of chalcogenides began in the early 2000s,^[^ ^64^, ^65^ ^]^ with the discovery of exceptional photocatalytic properties in materials like CdS and ZnS, sparked interest in water treatment.^[^ ^66^, ^67^ ^]^ This period also saw the development of binary and ternary chalcogenide systems, offering improved stability and efficiency.^[^ ^68^, ^69^ ^]^ Between the 2000s and 2010s (Figure 1), the production of chalcogenide‐based materials witnessed remarkable growth, translating to the development of narrow bandgap chalcogenides such as CdS, Bi₂S₃, and Cu₂S, facilitating the solar‐driven photocatalysis involving water splitting and pollutant degradation. The CdS and ZnS were employed in the photodegradation processes of dyes and other organic contaminants, while Bi₂S₃ and Sb₂S₃ demonstrated their potential for solar‐driven water purification and the mitigation of toxic metals. This era also witnessed the development of chalcogenide‐based sensors for gas detection.

A significant breakthrough came with the mid‐2000s isolation of 2D transition metal dichalcogenides (TMDs), such as MoS_2_, which revolutionized environmental catalysis due to their unique electronic structures, high surface area, and abundant active sites.^[^ ^70^, ^71^ ^]^ Since the mid‐2010s to date, it can be termed as the era of nanotechnology and has seen the development of nanostructured and hybrid systems. The synthesis of chalcogenide nanomaterials, such as nanosheets, quantum dots, etc., with significantly improved surface area, reactivity, and selectivity has been created. In addition, the development of Z‐scheme and type‐II heterojunctions has enhanced the charge separation (Figure 1). The incorporation of carbon materials, such as graphene and carbon nanotubes, significantly enhanced the efficacy of environmental remediation efforts. Different sulfide‐based adsorbents for the removal of heavy metal ions have been investigated. Also, the emergence of selenides and tellurides has been examined for their roles in redox reactions and their potential applications in sensing technologies. Recent years have shifted focus toward sustainable, earth‐abundant alternatives, moving away from toxic elements like cadmium and lead, driven by advancements in characterization techniques and theoretical insights into structure–property relationships.^[^ ^72^, ^73^ ^]^ The field now emphasizes rational design, combining computational modeling and experimental methods to enhance stability, efficiency, and scalability of chalcogenide materials for practical environmental applications.^[^ ^74^ ^]^

### 2.2 Properties and Applications of Metal Chalcogenides

**Figure 2:**
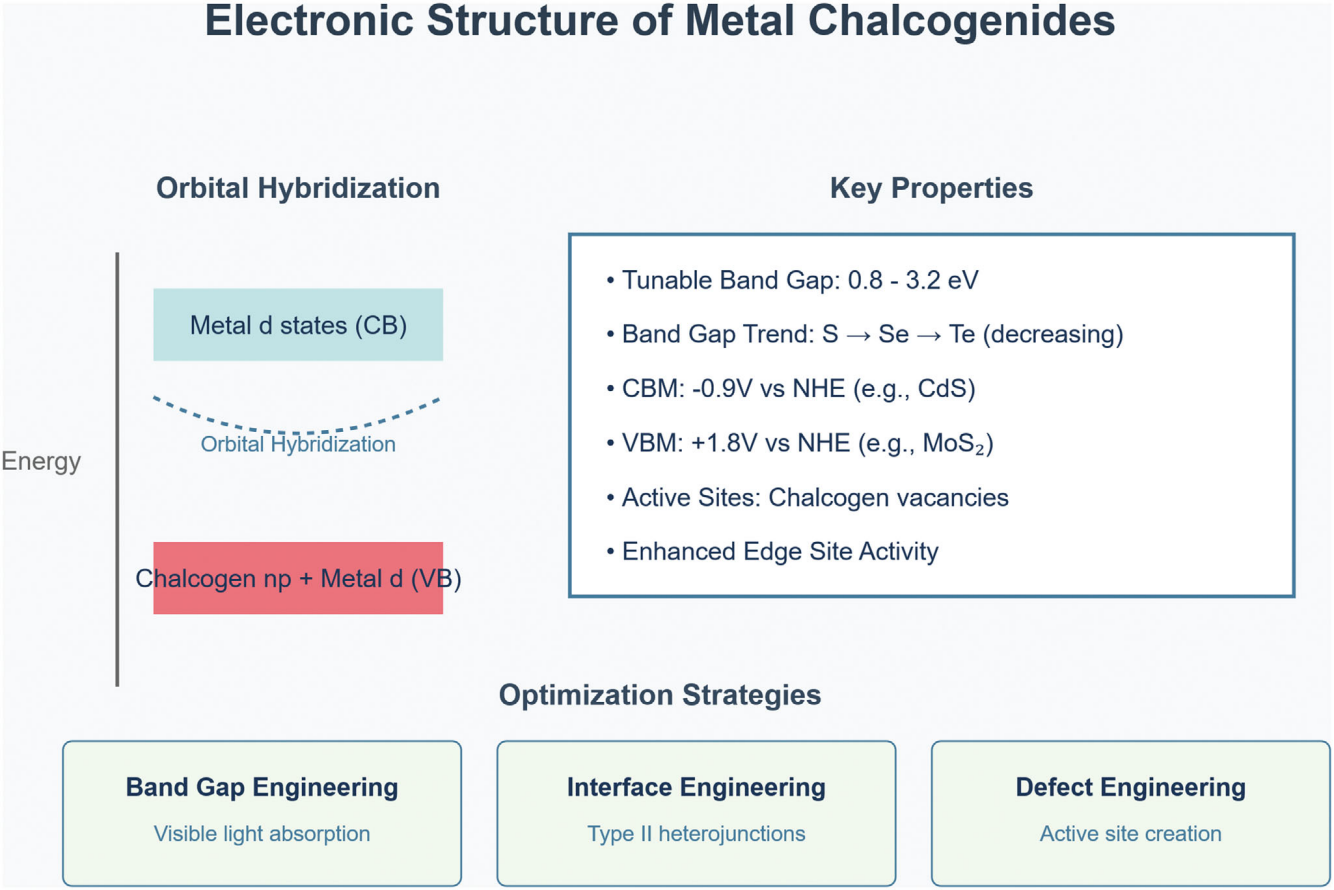
Electronic Structure and Properties of Metal Chalcogenides.

Metal chalcogenides demonstrate several fundamental properties that make them unique in materials science. At their core, these materials function as semiconductors with a bandgap energy typically ranging from 0.8 to 3.2 eV, following a systematic decrease from sulfur to selenium to tellurium compounds^[^ ^75^ ^]^ (**Figure** **2**), characterized by weak attraction forces maintained through Van der Waals interactions. Their bonding structure exhibits both homopolar and heteropolar characteristics, following the 8 N bonding rule.^[^ ^76^ ^]^ The orbital hybridization diagram as shown in Figure 2, reveals the complex interplay between metal d states in the conduction band and the hybridized chalcogen np and metal d states in the valence band, forming the electronic structure foundation of chalcogenide materials.^[^ ^76^ ^]^ The energetic positioning of the bands, characterized by a conduction band minimum at −0.9 V versus NHE (as seen in CdS) and a valence band maximum at +1.8 V versus NHE (exemplified by MoS₂), creates optimal conditions for various applications.^[^ ^77^ ^]^ The presence of chalcogen vacancies functions as catalytically active sites, particularly at edge positions,^[^ ^78^, ^79^ ^]^ while the material's performance can be strategically enhanced through three distinct approaches: band gap engineering to optimize visible light absorption,^[^ ^79^ ^]^ interface engineering to develop type II heterojunctions,^[^ ^80^ ^]^ and defect engineering to create and control active sites for improved functionality.^[^ ^81^ ^]^

As illustrated in Figure 2, metal chalcogenides represent a significant focus of research within the realm of ultrathin materials. For example, single‐layer MoS_2_, characterized by its direct band gap of ≈1.9 eV, has garnered significant attention in the realms of physics and nanoelectronics.^[^ ^82^, ^83^ ^]^ Its properties make it an excellent complement to semimetallic graphene and insulating h‐BN monolayers, serving as a vital component in the development of flexible 2D electronics. Their distinct structural, electronic, and optical characteristics are evident, including spontaneous rippling in free‐standing monolayers, notable modifications in the electronic band structure, substantial spin–orbit splitting, and increased photoluminescence. The majority of those properties are inherent to the monolayers and are notably lacking in two‐layer stacks of the same 2D crystal.^[^ ^83^ ^]^ For instance, single‐layer MoS_2_ exhibits characteristics of a direct band gap semiconductor, featuring a spin–orbit splitting of 150 meV within the valence band.^[^ ^82^, ^83^ ^]^ In contrast, the bilayer form of this material behaves as an indirect band gap semiconductor, lacking any discernible spin–orbit splitting. These attributes have been experimented and align remarkably well with density‐functional theory.^[^ ^82^, ^83^ ^]^ This account presents theoretical investigations concerning a subset of metal dichalcogenides characterized by the formula MX_2_, where M represents either Mo or W and X denotes Se or S, commonly known as “MoWSeS materials.” The findings regarding the electronic structure, spin–orbit coupling, quantum confinement, adjustments in electronic properties, and spontaneous monolayer rippling under the influence of an external electric field have been reported.^[^ ^82^, ^84^, ^85^, ^86^, ^87^, ^88^ ^]^ Although all materials within the MoWSeS family exhibit similar qualitative characteristics, their specific values vary significantly. For instance, the spin–orbit splitting in WSe_2_ attains a value of 428 meV, which is nearly three times greater than that of MoS_2_.^[^ ^82^, ^83^ ^]^

The fundamental nature of chalcogens themselves contributes significantly to their compound properties. Despite their varying atomic sizes, all chalcogens possess six valence electrons. Physical properties such as boiling point, melting point, density, and atomic radius increase proportionally with atomic weight. The elements show varying metallic characteristics, with oxygen, selenium, and sulfur classified as non‐metals, while polonium and tellurium function as semimetals capable of conducting electricity. A notable distinction exists between crystalline and non‐crystalline forms, where crystalline chalcogenides display specific ordered features while their non‐crystalline counterparts lack long‐range order and exhibit distinctive energy bandgaps.^[^ ^89^ ^]^

Various types of metal halides and chalcogenides are under investigation and have been categorized into main group, transition group, and rare earth metal chalcogenide halides, with additional subdivisions based on the chemical and physical composition.^[^ ^90^ ^]^ All chalcogens demonstrate high reactivity with alkaline earth metals and exist in ionic forms within metallic ores. Chalcogens can be divided into two distinct groups: light and heavy. The light chalcogens—oxygen and sulfur—are vital to life processes and are naturally present in our bodies. In contrast, heavy chalcogens such as selenium, tellurium, and polonium can be harmful to living organisms due to their toxic properties. **Table** **1** provides a summary of the properties of chalcogenide materials based on their category. These exceptional properties render them suitable for numerous applications. They have garnered considerable attention in various fields, including water/wastewater treatment technologies, electronic devices, including transistors, energy storage, and sensors. Chalcogenide nanostructures exhibit unique thermal characteristics, making them particularly advantageous for thermoelectric applications.

**Table 1:** Properties of Chalcogenide Materials.

Property category	Property	Alkali/alkaline‐earth chalcogenides^[^ ^91^ ^]^	Transition metal chalcogenides^[^ ^92^ ^]^	Nanostructured chalcogenides^[^ ^93^ ^]^	Chalcogenide glasses^[^ ^94^ ^]^
Structural properties	Crystal system	Primarily cubic structures, such as rock salt (NaCl‐type) and antifluorite.	Layered structures often have hexagonal or octahedral coordination.	Variable: includes 1D chains and layered structures.	Amorphous.
Electronic properties	Band gap	Wide band gaps, typically insulating.	Narrow band gaps can be semiconducting or metallic.	Tunable band gaps depending on size and structure.	Generally, exhibit mid‐range band gaps; can be semiconducting.
Optical properties	Transparency range	Transparent in the visible range.	Absorption in the visible to near‐infrared range.	Size‐dependent optical properties; quantum confinement effects.	Transparent in the infrared range.
Mechanical properties	Hardness	Generally soft, low hardness values.	Variable: Some are relatively hard due to strong covalent bonding.	Enhanced mechanical properties due to nano‐structuring.	Typically, brittle.
Thermal properties	Melting point	Moderate to high melting points.	Variable: Some decompose before melting.	Lower melting points due to the nanoscale size.	Lower melting points compared to crystalline counterparts.

The transformation of chalcogenides to nanoscale dimensions has opened new frontiers in their application potential. When reduced to the nanoscale, these materials exhibit novel physical and chemical features attributed to quantum size effects. Their significantly enhanced surface area compared to bulk forms makes them particularly valuable for environmental applications, as it substantially improves interaction between the device and the surrounding medium. This property, combined with their inherent characteristics, has led to their increasing adoption in various applications, including water purification and energy devices. A study by Cui and colleagues examined both the electrical and optical distinctiveness of “graphene‐like gallium nitride doped with transition metal dichalcogenide heterostructures”, demonstrating enhanced efficacy in water separation.^[^ ^95^ ^]^ Nanostructured metal chalcogenides demonstrate superior efficacy compared to their bulk counterparts due to several key factors: enhanced specific surface areas with increased active sites, extended cycle longevity, improved electrical conductivity, and reduced electron transport path lengths.^[^ ^96^, ^97^ ^]^ In addition, Zhao and colleagues examined identical optical spectra and electronic character for electron‐doping strategies in dichalcogenides.^[^ ^98^ ^]^

Metal carbonyl clusters within chalcogenides present interesting properties of their own. These structures demonstrate superior redox properties compared to highly valent organometallic complexes, primarily due to the introduction of main group elements or metal pieces achieved through redox potential shift. They exhibit semiconducting properties characterized by pie–pie interaction. While anion odd‐electron metal carbonyl clusters show paramagnetic behavior due to inherent instability, the paramagnetic characteristics of even‐electron species remain less extensively studied.^[^ ^99^ ^]^ Recent research has demonstrated their exceptional performance in practical applications. For instance, the combination of ferrous ferric oxide and molybdenum disulfide has shown superior adsorption capabilities for methylene blue compared to conventional nanomaterials, primarily due to their elevated surface area.^[^ ^100^ ^]^ In water treatment applications, chalcogenides have demonstrated remarkable efficiency. Studies have shown impressive results in antibiotic removal, with titanium dioxide‐cadmium sulfide compositions achieving up to 98.1% elimination of tetracycline hydrochloride.^[^ ^101^ ^]^ Similarly, combinations of silver indium disulfides with titanium dioxides have demonstrated over 95% removal efficiency for doxycycline.^[^ ^102^ ^]^ Advanced materials development continues to expand its potential, particularly through integration with graphene‐like structures and various metal halide combinations. These materials are increasingly being recognized as preferred choices for solar absorber and device configurations, marking their growing importance in sustainable technology applications.^[^ ^96^ ^]^

## 3 Synthetic Methods of Chalcogenide Materials

The fabrication of chalcogenide materials with optimal characteristics for specific applications is often challenging due to the instability of precursors and the intricate coordination, polymerization, and condensation chemistry involved in metal‐chalcogenide compounds, which complicates achieving desired morphologies and functionalities.^[^ ^103^, ^104^ ^]^ These challenges have been partially addressed through synthesis methods that regulate interactions between precursors and structural components, enabling the production of chalcogenide materials with diverse structures. Generally, these synthesis strategies can be categorized into the following

### 3.1 Ball Milling

This is a simple and economical mechanochemical technique effectively employed for the synthesis of chalcogenides, including sulfides and selenides.^[^ ^105^, ^106^, ^107^, ^108^, ^109^, ^110^, ^111^, ^112^ ^]^ This technique entails a combination of the metal or precursor with the chalcogen or its precursors via ball milling. An alloy is produced between the metal and the chalcogen in collisions with high energy within the ball mill. Nonetheless, regulating the resultant phase of the alloy is challenging.^[^ ^108^ ^]^ Furthermore, as the duration of the impact extends, the product gets polluted by metallic spheres and the container.^[^ ^108^, ^113^ ^]^ It should be noted that the ball milling is frequently succeeded by annealing to enhance crystallization and diminish contaminants and the product's phases.^[^ ^114^ ^]^

**Figure 3:**
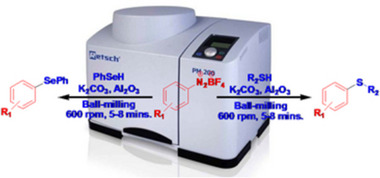
Ball‐milling synthetic process of unsymmetrical selenides and sulfides. Reproduced with permission.^[^ ^106^ ^]^ Copyright 2015, Semantic Scholar.

A process free of transition metals and solvents for the reaction of aryl diazonium tetrafluoroborate with thiol or selenol on an alumina surface has been developed via ball‐milling at room temperature for unsymmetrical aryl‐alkyl or diaryl  selenides and sulfides (**Figure** **3**). This produced a diverse array of functionalized aryl‐alkyl or diaryl selenides and sulfides having a higher purity and good yield within a short reaction time of 5–8 min.

**Figure 4:**
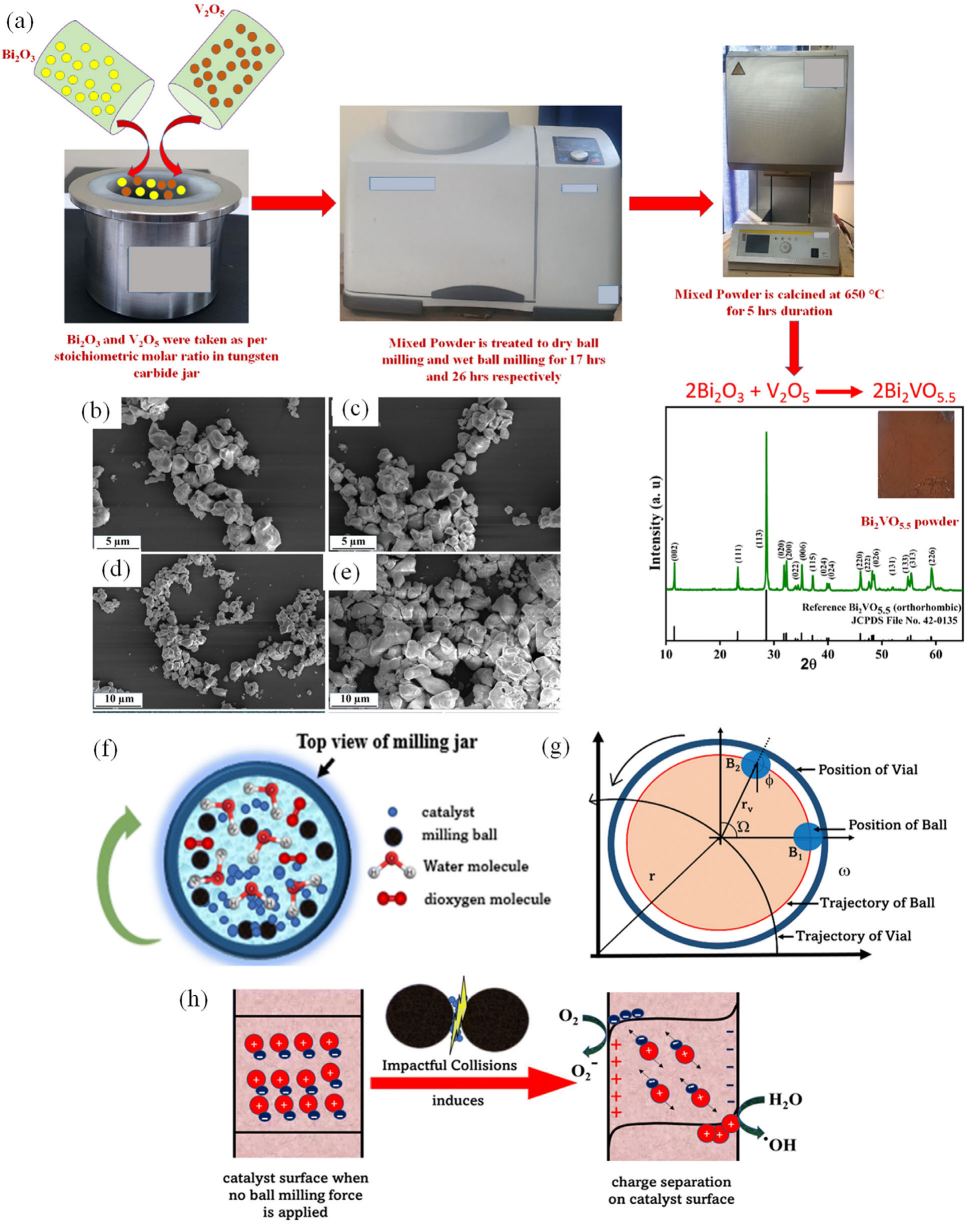
a) Preparation process of BV powder using balling milling technique, SEM images of b–d) BV and e) BiV samples. Reproduced with permisison.^[^ ^115^ ^]^ Copyright 2023, Nature. f) Top depiction of the ball milling jar, g) the path of the balls within the milling jar, and h) a schematic illustration demonstrating the generation of reactive species when SrTiO_3_ becomes entrapped between two balls during a collision. Reproduced with permission.^[^ ^116^ ^]^ Copyright 2024, RSC.

Mechanochemical ball milling process, which was succeeded by thermal treatment at 650 °C for a duration of 5 h, has been reported to effectively yield a single‐phase Bi_2_VO_5.5_ powder (**Figure** **4**a–e).^[^ ^115^ ^]^ This was employed for photocatalytic activity associated with the degradation of methylene blue dye, demonstrating 63% efficiency. Similarly, the capability of ball milling to enhance materials with low piezoelectric coefficients for catalysis has been demonstrated by the mechanocatalytic activity created in SrTiO_3_ nanoparticles for degrading methylene blue dye. Parametric tests conducted to examine the impact of several process variables, including dose, dye concentration, ball milling speed, and the quantity of milling balls, showed that the as‐obtained SrTiO_3_ exhibited just 12% deterioration during one hour when compared with the ultrasonication method.^[^ ^116^ ^]^ Figure 4f–h illustrates the schematic interpretation of the mill jar's top view, the trajectory of balls within the milling jar, and the pictorial depiction of reactive species formation during collisions.

### 3.2 Chemical Vapor Deposition

Chemical vapor deposition (CVD) method has been shown as an efficient approach for the synthesis of 2D materials possessing impressive crystal quality, particularly for producing uniform thickness, wafer‐scale, or larger single‐crystal domain size. Typically, a salt‐assisted CVD technique has demonstrated efficacy in lowering the higher melting point of metal‐related precursors, thereby reducing nucleation density, while boosting the reactivity rates on the solid templates. Nonetheless, the precise functions of alkali metal(s) and halide constituents remain ambiguous. In contrast to the basal plane, the catalyst edges may include greater numbers of active sites; hence, additional approaches including atomic layer and electrochemical deposition are employed to create a thin catalyst layer possessing a greater number of edges.^[^ ^117^, ^118^ ^]^ At the atomic level, the preparation techniques can be refined to regulate the catalyst distribution on the supporting materials. For instance, a single atomic active site was synthesized by precursor‐ and condition‐controlled CVD.^[^ ^119^, ^120^ ^]^ In this method, the deposition of the elements onto the heated substrate through the chemical reactions occurring in the vapor phase. Notably, the CVD technique contains several benefits, including the ability to coat surfaces with intricate geometries and uneven morphologies, a rapid rate of deposition, and no requirement for a high vacuum.^[^ ^121^ ^]^ However, the CVD method has the challenges associated with the use of corrosive and toxic chemical vapor at high temperatures.

**Figure 5:**
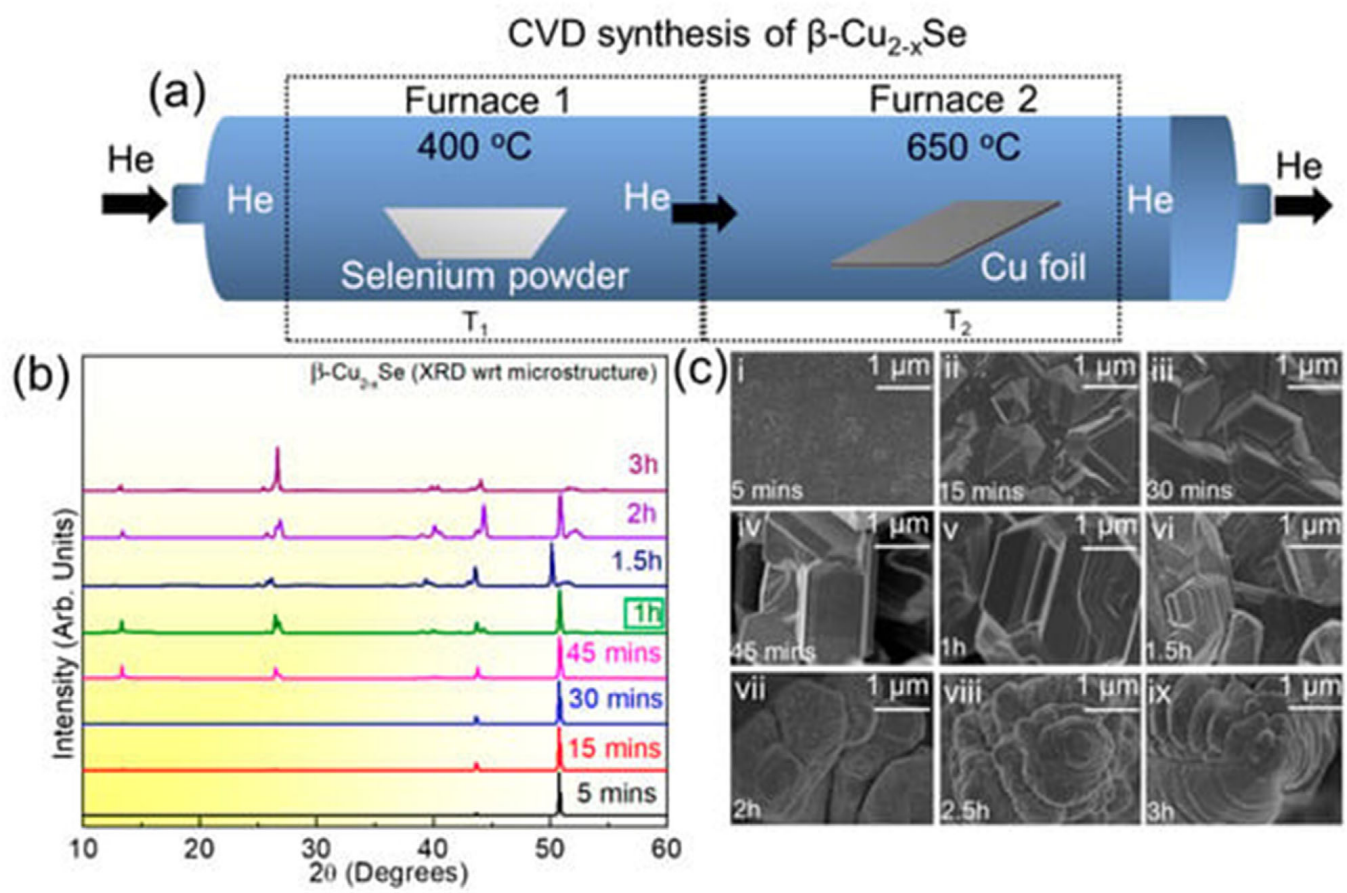
a) Synthesis and optimization of Cu_2_‐xSe on copper foil using the CVD technique. b) XRD illustrating the Cu_2_‐xSe on copper foil; c) HRSEM depicting the development of microstructural elements and the formation of Cu_2_‐xSe over varying reaction times (5 min to 3 h) between the etched copper foil and elemental selenium. Reproduced with permission.^[^ ^122^ ^]^ Copyright 2024, MDPI.

Srijith et al. produced copper selenide (β‐Cu_2_−xSe) using CVD method (**Figure** **5**) and identified it as an efficient photocatalyst for degrading tetracycline hydrochloride.^[^ ^122^ ^]^ The photocatalyst exhibited a remarkable degrading efficiency of 98% with a reaction rate constant of 3.14 × 10^−2^ per min.

### 3.3 Solvothermal/Hydrothermal Technique

The solvothermal autoclave method is often employed to mitigate the elevated temperatures and significant interfacial energy disparity between the particle and substrate.^[^ ^123^, ^124^ ^]^ The hydrothermal approach employs a solvent within a pressured Teflon vessel using a stainless‐steel pressure reactor (autoclave). An autoclave system follows by heating to a temperature beyond the solvents' saturation temperature, therefore elevating the pressure within the reactor. The photocatalyst is produced under sustained higher‐temperature and higher‐pressure environments. The pressure within the reactor is then contingent upon the volume and types of liquid employed by the Teflon containers, in addition to the temperature of operation.^[^ ^59^, ^124^, ^125^ ^]^

In an unregulated environment, crystals initiate as seed that subsequently aggregate, optimizing stability and decreasing the surface area, mostly resulting in spherical forms through Ostwald ripening.^[^ ^59^, ^126^ ^]^ It should be noted that modification can be achieved by adjusting the nuclear constituents and implementing growth‐direction controls using additives. Moreover, the interior layers of a defined shape may ascend to its surfaces and recrystallize, resulting in porous structures.^[^ ^127^ ^]^

**Figure 6:**
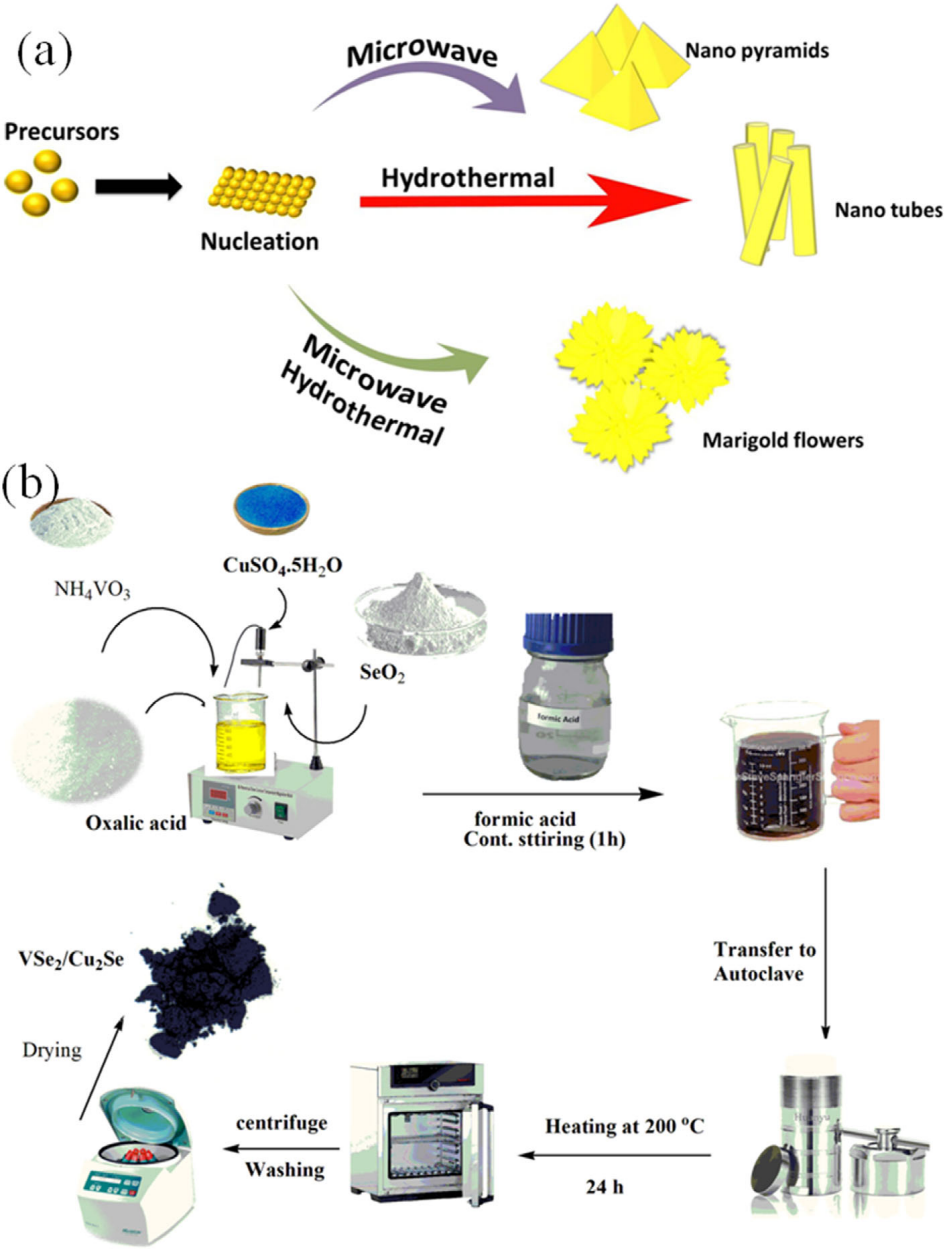
a) Schematic illustration of various morphologies in CdIn_2_S_4_. Reproduced with permission.^[^ ^128^ ^]^ Copyright 2010, RSC. b) Synthetic route of VSe_2_@Cu_2_Se nanocomposites. Reproduced with permission.^[^ ^129^ ^]^ Copyright 2023, Physica Scripta.

Cubic spinel CdIn_2_S_4_ has been synthesized using including solvothermal and hydrothermal methods.^[^ ^128^ ^]^ The as‐produced sample using the C_2_H_5_OH‐mediated solvothermal method showed nanotube morphology, whereas the sample produced using the water‐mediated hydrothermal method had puffy marigold‐like morphology. The optimization during the synthetic settings produced a range of CdIn_2_S_4_ morphologies, including nanopyramid and flower cluster (**Figure** **6**). The photodegradation methylene blue dye demonstrated 30% over CdIn_2_S_4_ compared to that of CdS. The straightforward, inexpensive, and ecologically benign one‐pot hydrothermal process has been used to synthesize VSe_2_, Cu_2_Se, and VSe_2_@Cu_2_Se nanoparticles.^[^ ^129^ ^]^ They showed a pure crystalline cubic crystal structure, and the crystallite sizes vary according to their composition with VSe_2_@Cu_2_Se composites demonstrated 25.9 nm, which was less than that of either component in their native forms

The solvothermal synthesis of a flower‐like CoS hollow sphere has be investigated using CTAB as microwave absorber.^[^ ^127^ ^]^ Under comparable preparation circumstances, it was discovered that this microstructure differed considerably from the CoS nanoparticle made without the addition of CTAB.

### 3.4 Co‐Precipitation

**Figure 7:**
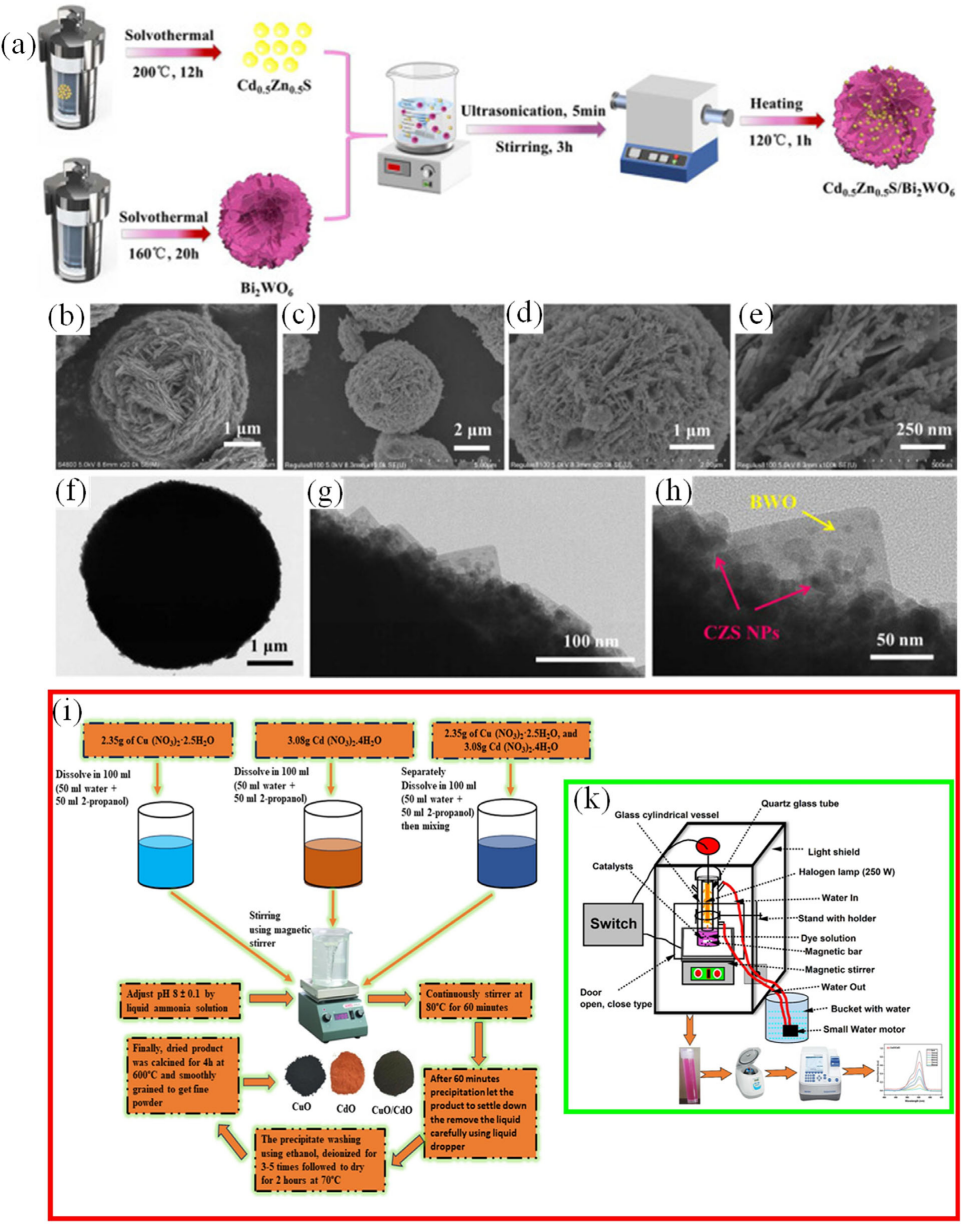
a) “Synthesis of Cd_0.5_Zn_0.5_S/Bi_2_WO_6_ (CZS/BWO) S‐scheme heterojunction. SEM images of b) BWO and c–e) 1.0CZS/BWO; f–h) TEM images”. Reproduced with permission.^[^ ^134^ ^]^ Copyright 2023, Elsevier. i) The synthesis of CuO and CdO nanoparticles, and CuO/CdO nanocomposite j) Photodegradation setup for RhB dye. Reproduced with permission.^[^ ^135^ ^]^ Copyright 2023, Elsevier.

This is a straightforward technique employed for the synthesis of metal sulfides. This technique needs a solvent capable of dissolving sulfide precursors and metal salts. The shape and dimensions of a metal sulfide particle synthesized by the co‐precipitation process are regulated through various additives, including ligands, chelating agents, scaffolds, and surfactants. The pH, mixing composition, and temperature of the reaction significantly influence the reaction yield. Nonetheless, the challenge of eliminating such chemicals is a primary drawback of the co‐precipitation strategy. Nevertheless, it can generate nanoscale materials, for example, 0D particles.^[^ ^130^, ^131^, ^132^, ^133^ ^]^ For example, an innovative Cd_0.5_Zn_0.5_S/Bi_2_WO_6_ S‐scheme heterojunction has been constructed through integrating Cd_0.5_Zn_0.5_S nanoparticle onto a Bi_2_WO_6_ microsphere through a via precipitation method (**Figure** **7**). The S‐scheme charge transfer mechanism significantly enhances spatial separation and retention of higher‐energy electron and hole pairs on Cd_0.5_Zn_0.5_S and Bi_2_WO_6_, respectively. The as‐obtained Cd_0.5_Zn_0.5_S/Bi_2_WO_6_ heterojunction demonstrates remarkable efficacy in photodegradation of tetracycline under visible light.^[^ ^134^ ^]^

The synthesis of hexagonal grain having a nanoporous structure has been reported by combining the non‐ionic surfactant Triton X‐100 mix with CuO/CdO.^[^ ^135^ ^]^ Here, the cubic and monoclinic phases were observed to be displaced in the pristine CdO and CuO, respectively. The photodegradation of RhB dye translated pristine of 71.42% and 77.83% over CuO and CdO, respectively while that of CuO/CdO composite demonstrated a 94% efficiency.^[^ ^135^ ^]^

### 3.5 Template‐Directed Methods

Although numerous synthetic methodologies have been established for the fabrication of mesoporous materials in recent decades, template‐assisted methods continue to be the most appealing and dependable approach for the design and development of mesostructured functional materials. In template‐assisted synthesis, the “template” is an essential component that denotes any entity exhibiting nanostructured characteristics, which can act as a scaffold to direct the formation of a mesoporous nanostructure with diverse morphologies and geometries. The dimensions, shape, and charge distributions of the template substantially influence its structural‐guiding characteristics. Template‐assisted tactics can be broadly categorized into two primary types based on the template's characteristics: 1) soft template and 2) hard template approaches.^[^ ^136^ ^]^

#### 3.5.1 Soft Template Method

**Figure 8:**
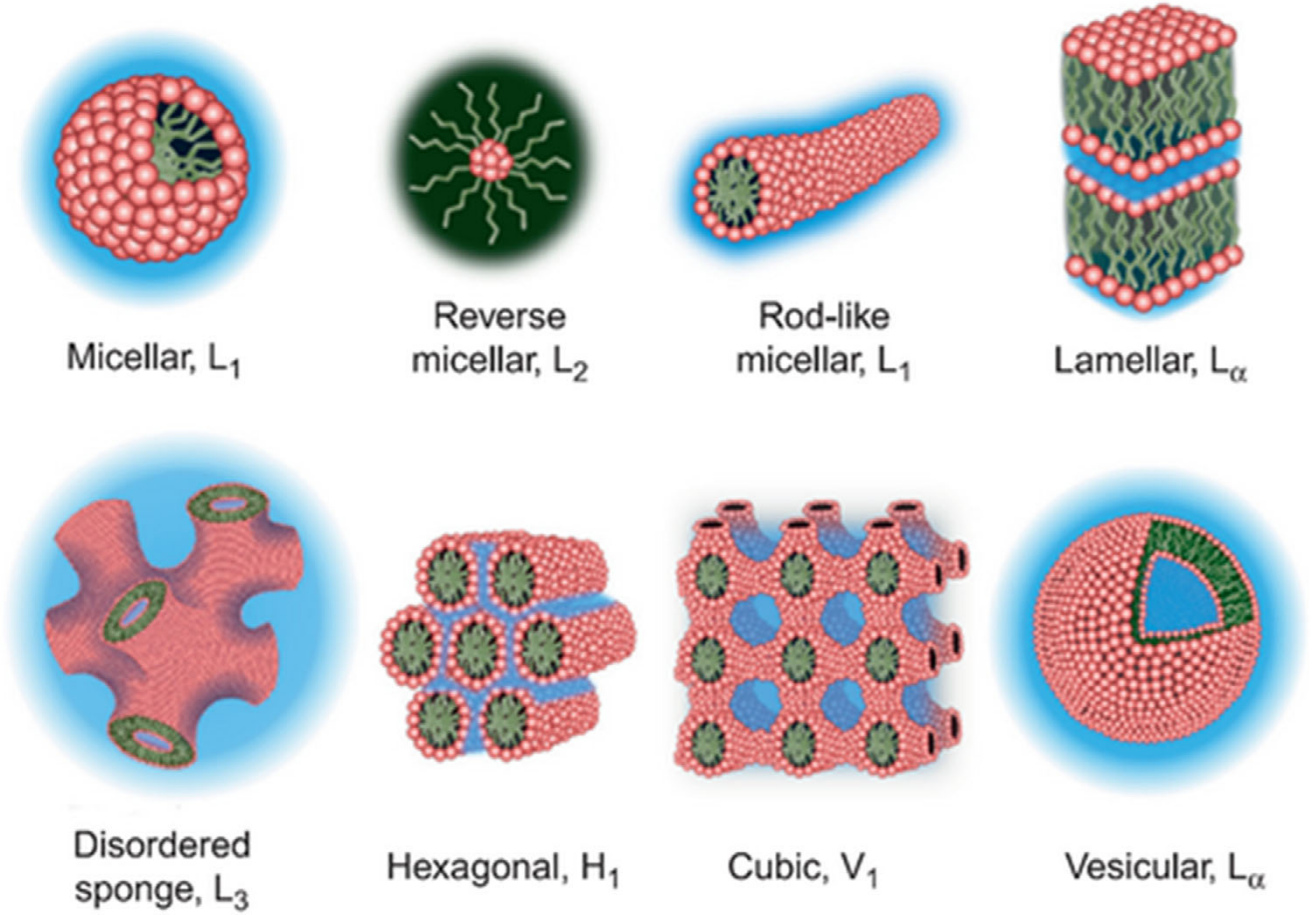
A typical example of self‐assembled liquid phase crystal architectures. Reproduced with permission.^[^ ^140^ ^]^ Copyright 2014, Elsevier.

The soft‐template approach utilizes soft‐matter supramolecular aggregates, such as block copolymers or amphiphilic surfactant molecules, to generate mesopore organization through co‐assembly having inorganic/organic guest species.^[^ ^137^, ^138^ ^]^ Due to the inherent hydrophobic effects and amphiphilic properties, these amphiphilic molecules self‐assemble into micellar structures, including a non‐polar (hydrophobic) core and an outside layer of a polar group, while distributed among the polar media (ethanol or H_2_O). Micelle is the most basic self‐assemblage structure, created through a precise concentration threshold, referred to as the “critical micelle concentration (CMC)”, is exceeded. As the concentration surpasses the CMC, alternative aggregate structures become thermodynamically advantageous, contingent upon the geometric attributes of the specific amphiphile. As presented in **Figure** **8**, these include extended micelle (“rodlike” “wormlike” or “cylindrical” structure), sheet‐like (lamellar) configurations, and various liquid phase crystalline exhibiting longer range mesoscopic orderly (cubic, hexagonal‐packed, and sponge phase).^[^ ^139^, ^140^ ^]^

The capacity of an amphiphilic organic molecule to generate micelle and liquid‐crystalline mesophase is a critical factor influencing the synthesis of ordering mesostructured materials. Consequently, these compounds are referred to as structure‐directing agents (SDA). Upon the existence of guest species frameworks, organic SDA co‐assembles with organic or inorganic components to create orderly hybrid mesostructured materials. The interaction between the organic molecule template and guest species is regarded as the primary driving force necessary for achieving an orderly template mesophase. The interaction mostly consists of weak noncovalent bonds, including hydrogen bonding and electrostatic interactions or Van der Waals.^[^ ^138^, ^141^, ^142^, ^143^, ^144^ ^]^

**Figure 9:**
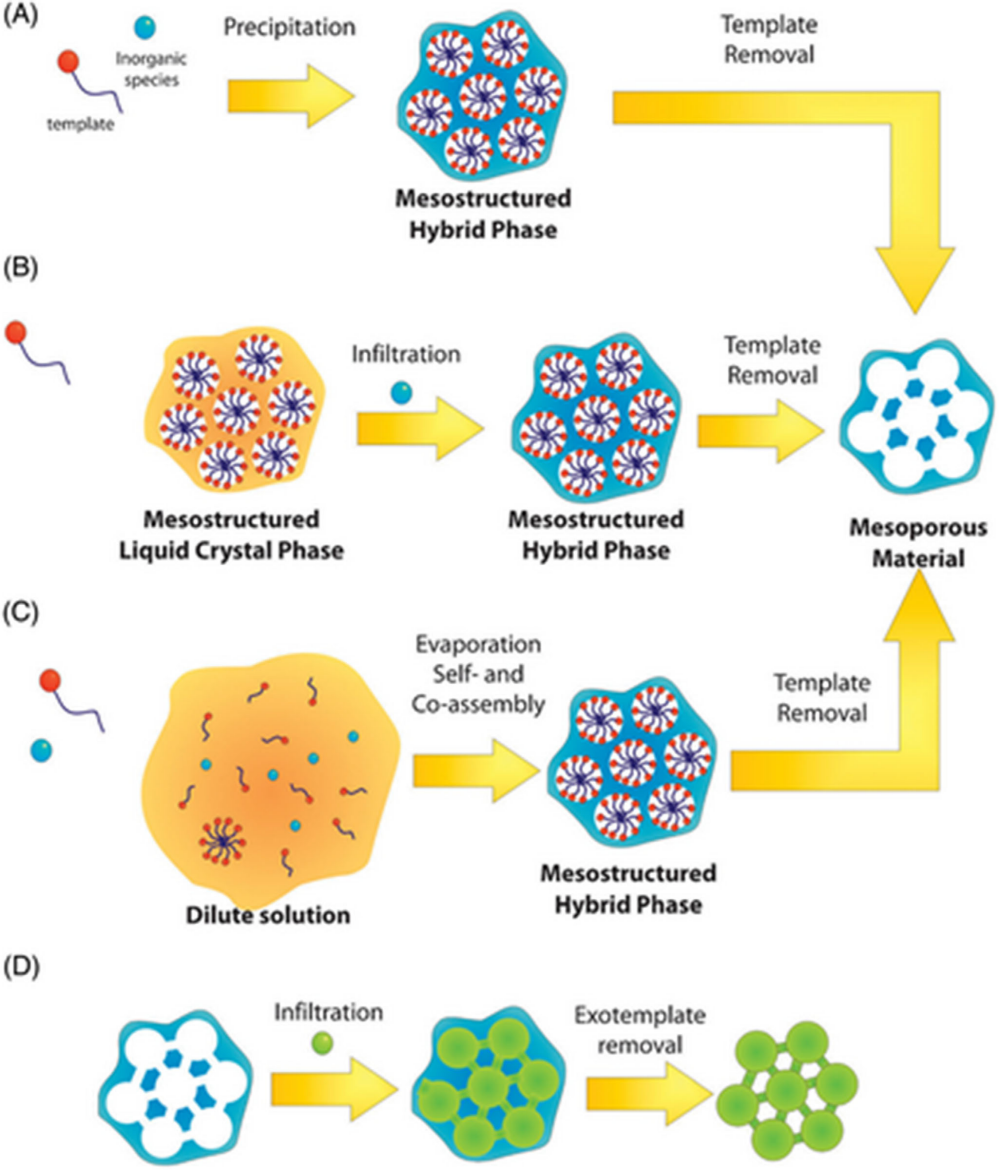
Schematic representation of the primary synthesis pathways for mesoporous materials. a–c) Soft template techniques: a) CSA, b) TLCT, and c) EISA. d) Hard‐templating technique. Reproduced with permission.^[^ ^145^ ^]^ Copyright 2011, RSC.

Ultimately, following the elimination of an organic template through pyrolysis, calcination, solvent extraction, or ion‐exchange, the resultant ordered mesoporous materials (with open pores) could be acquired, exhibiting a pore architecture akin to that of the “liquid‐crystal mesophase”. As presented in **Table** **2**, regarding this overarching soft template strategy, three principal paths were put forward for the efficient fabrication of orderly mesostructured nanomaterials: “cooperative self‐assembly (CSA)”, “true liquid‐crystal templating (TLCT)”, and “evaporation‐induced self‐assembly (EISA)” (**Figure** **9**).

**Table 2:** Comparison of synthetic approaches for chalcogenide materials.

Approaches		Fundamental principles	Pros	Cons
	CSA	i) Cooperation assemblage of inorganic and organic constituents ii) Development of mesostructures governed by intermolecular forces, inorganic–organic interaction, and hydrophobic effects	i) Controlled mesostructure and pore dimensions	i) Mesostructural development is acutely responsive to the reaction circumstances. ii) Amorphous/comparatively low crystalline substances
	TLCT	i) Introducing inorganic constituents to an established liquid crystal mesophase ii) Mesostructural development regulated by the rates of inorganic dispersion and condensation	i) Predictable mesostructure and pore geometries derived from established liquid‐crystal mesophase ii) Well‐organized mesostructure	i) Restricted regulation of inorganic diffusion and condensation
Soft‐templating method	EISA	i) Co‐assembly of organic and inorganic constituents under solvent evaporation conditions ii) Formation of mesostructures regulated by organic–inorganic interaction and rates of condensation	i) Minimal surfactant concentration ii) Enhanced regulation of inorganic condensation‐polymerization iii) Readily processable mesostructure in diverse forms (thin films, powders, gels)	i) Necessitates an additional processing step ii) More challenging to acquire a highly organized mesostructure
Hard‐templating techniques		i) Introducing inorganic constituents into pre‐existing mesoporous materials (rigid templates) ii) Formation of mesostructure regulated by the diffusion and condensation of inorganic precursors	i) Minimal susceptibility to reaction conditions ii) Highly organized mesostructure iii) Highly crystalline materials	i) Employs prefabricated rigid templates ii) Restricted control over pore dimensions and structure iii) Necessitates many processing stages (time‐intensive) iv) Small‐scale manufacturing

#### 3.5.2 Hard Template Method

The synthesis of orderly mesoporous inorganic framework via the aforementioned soft‐template methods (EISA, TLCT, and CSA) necessitates meticulous regulation of numerous chemical and physical parameters, including inorganic/organic interactions, template concentration, condensation rate, crystallization, humidity, temperature, and evaporation conditions. An alternate approach known as “hard‐templating” was recently developed to overcome the complexities and limits of soft‐template synthesis. This approach, referred to as “nanocasting” or “exotemplating”, utilizes porous substances as a “hard template” to produce duplicates.^[^ ^146^, ^147^, ^148^ ^]^ The rigid exotemplate typically consists of a systematically arranged mesoporous silica featuring interconnected pore architectures, exemplified by “Mobil Composition of Matter No. 48 (MCM‐48)”,^[^ ^149^ ^]^ “Santa Barbara Amorphous (SBA)‐15”,^[^ ^150^ ^]^ “Fudan University‐5 (FDU‐5)”,^[^ ^151^ ^]^ or “Korean Institute of Technology‐6 (KIT‐6)”,^[^ ^152^ ^]^ which is permeated with appropriate precursor(s) and subsequently passed through thermal or chemical treatment to yield the intended phases.

The precursor infiltration process may be reiterated as often as necessary to attain the suitable precursor loading required for the self‐support and rigidification of the last replica mesostructures. Upon reaching the requisite degree of solidifications, the exotemplates could be preferentially eliminated with sodium hydroxide (NaOH) or hydrofluoric acid (HF) solution to yield the final mesostructures, which serve as a negative reproduction of the porous hard template architecture (Figure 7d). Nanocasting technique is especially advantageous for the synthesis of mesostructuring carbon and TMOs, which are often challenging to produce by standard soft template methods.^[^ ^153^, ^154^, ^155^, ^156^ ^]^

Over the past decades, the hard‐template technique has demonstrated efficacy in producing chalcogenide materials, primarily utilizing mesoporous silica as a hard template to accomplish the requisite mesoporous structures. The conventional method entails the infiltration of an appropriate precursor into a rigid template, the creating the solid phase via thermochemical processing, and the extraction of a template through NaOH or HF etch to ultimately acquire the chalcogenide materials. Numerous studies have documented the successful synthesis of chalcogenide materials with diverse chemical compositions, such as  FeS_2_, CdS,  CdSe, ZnS, ZnSe, CdS_x_Zn_1−x_Se, CdS_x_Zn_1−x_S, CuS, Ag_2_S, POM/Ag_2_S/CdS, NiS_2_, CoS_2_, WS_2_, WSe_2_, MoS_2_, and MoSe_2_ utilizing the hard‐template technique.^[^ ^157^, ^158^, ^159^, ^160^, ^161^, ^162^, ^163^ ^]^

**Figure 10:**
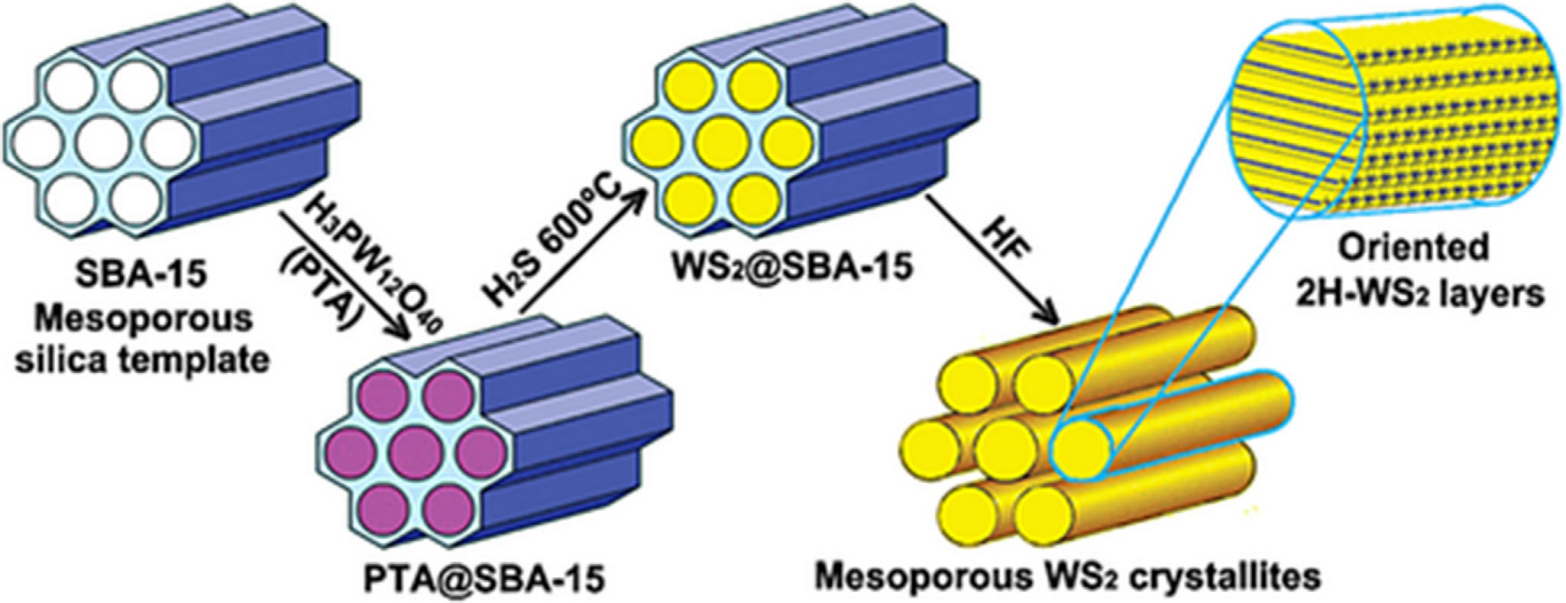
Illustration of a scheme demonstrating the hard‐template synthesis process utilizing SBA‐15 as the hard template, aimed at the fabrication of 2D hexagonally orderly mesoporous WS_2_. Phosphotungstic acid: H_3_PW_12_O_40_. Reproduced with permission.^[^ ^157^, ^159^ ^]^ Copyright 2023, Wiley; Copyright 2007, ACS.

Gao and co‐worker initially synthesized mesoporous CdS, MoS_2_ and WS_2_  with ordered 2D hexagonal (p6mm) and 3D bicontinuous cube systems, employing KIT‐6 and SBA‐15 mesoporous silica as hard template (**Figure** **10**).^[^ ^157^, ^159^ ^]^ Armatas group engineered multicomponent mesoporous POM/Ag_2_S/CdS heterostructure by two steps’ hard template and topo‐tactic ion‐exchange chemical method.^[^ ^160^ ^]^ Utilizing SBA‐15 as a rigid template alongside thiourea, cadmium nitrates, and other POM clusters.

A variety of mesoporous and orderly POM/CdS composites have been synthesized adopting SBA‐15 as a hard template with thiourea, cadmium nitrates, and various POM clusters (i.e., PMo_12_O_40_^3−^, SiW_12_O_40_^4−^, and PW_12_O_40_^3−^) as starting precursors. The final versions of POM/Ag_2_S/CdS mesoporous heterostructure were obtained through a partial cation‐exchange of Cd^2+^ ions with Ag+ ions. Another study described a universal “oxide‐to‐sulfide” technique for synthesizing mesoporous FeS_2_, NiS_2_, CoS_2_, and purportedly metal sulfide, by originally fabricating the corresponding metal oxides contained by the mesoporous KIT‐6 silica templates, followed by the conversion of the metal oxides to sulfides through thermal sulfurization.^[^ ^162^ ^]^

Nonetheless, while the hard‐templating method proves to be an efficient approach for synthesing of various mesostructured materials, especially those exhibiting high crystallinity, it is frequently associated with high costs, extended time requirements, and a lack of synthetic versatility, which restricts the ability to precisely control pore shapes and sizes. Furthermore, the absence of distinct pore morphology and consistency in the materials' porosity produced through various template‐free methods complicates the process of identifying and categorizing these materials within a specific class of porous materials.^[^ ^164^, ^165^, ^166^ ^]^

In contrast, the soft‐template technique, as a conventional “bottom–up” technique, offers distinct advantages due to the capacity to generate mesoporous materials characterized by well‐defined even pores with a significant level of regulation over both shape and pore size. Table 2 provides a concise assessment of different fabrication techniques. Consequently, soft templating continues to be the most appealing and widely adopted approach for the synthesis of mesoporous materials. Thus, this section primarily concentrates on a thorough analysis of advancements in the soft template‐assisted production of chalcogenide materials.

## 4 Recent Advances in the Transformative Potential of Chalcogenide Materials for Organic Pollutant Removal from Water/Wastewater

The organic degradation is one example of how the general public's growing concern and environmental preservation over the last few decades thereby attracting significant interest by various researchers. Materials belonging to the family of chalcogenide materials (II–VI and IV–VI) have bandgap that extend from the visible (e.g., CdTe, CdS, and CdSe) and UV (ZnSe and ZnS) regions to a near‐infrared (PbTe, PbS, and PbSe).^[^ ^96^ ^]^ Because of its acceptable bandgap, which covers almost the whole solar bandwidth, chalcogenide nanostructure materials have been employed for diverse environmental degradation and cleanliness applications. The unique forms, crystalline structures, composition, and size of chalcogenide materials cause them to display unique features. Typically, CuS has been reported to be the most researched and used because of its strong p‐type semiconductor properties and intriguing shape.^[^ ^96^ ^]^

Moreover, traditional treatment procedures are unable to deal with the serious environmental problems of water resource contamination caused by effluents from many sources, including industrial urban, and rural areas. These effluents include several toxic substances, including dyes, PPCP, and pesticides.^[^ ^167^, ^168^ ^]^ Admittedly, it is crucial to provide the groundwork for a sustainable future by establishing ecofriendly technology. However, tackling these concerns is challenging to both environmentalists and researchers. Accordingly, it is critical to enhance these technologies by creating new useful materials.

A variety of surface or interface‐related operations, including catalysis, adsorption, and separation, can be greatly enhanced by using chalcogenide materials. This is particularly true for new environmental applications, thanks to their distinctive structural attributes and distinctive physicochemical and electrical properties. They also have important attributes such as large pore volumes, tunable bandgaps, and consistent mesopore morphology which are required by efficient materials for environmental remediation. Therefore, this section shall focus on the recent progress involving the utilization of chalcogenides for removing dyes, PPCP, and pesticides from water/wastewater.

### 4.1 Dyes

**Figure 11:**
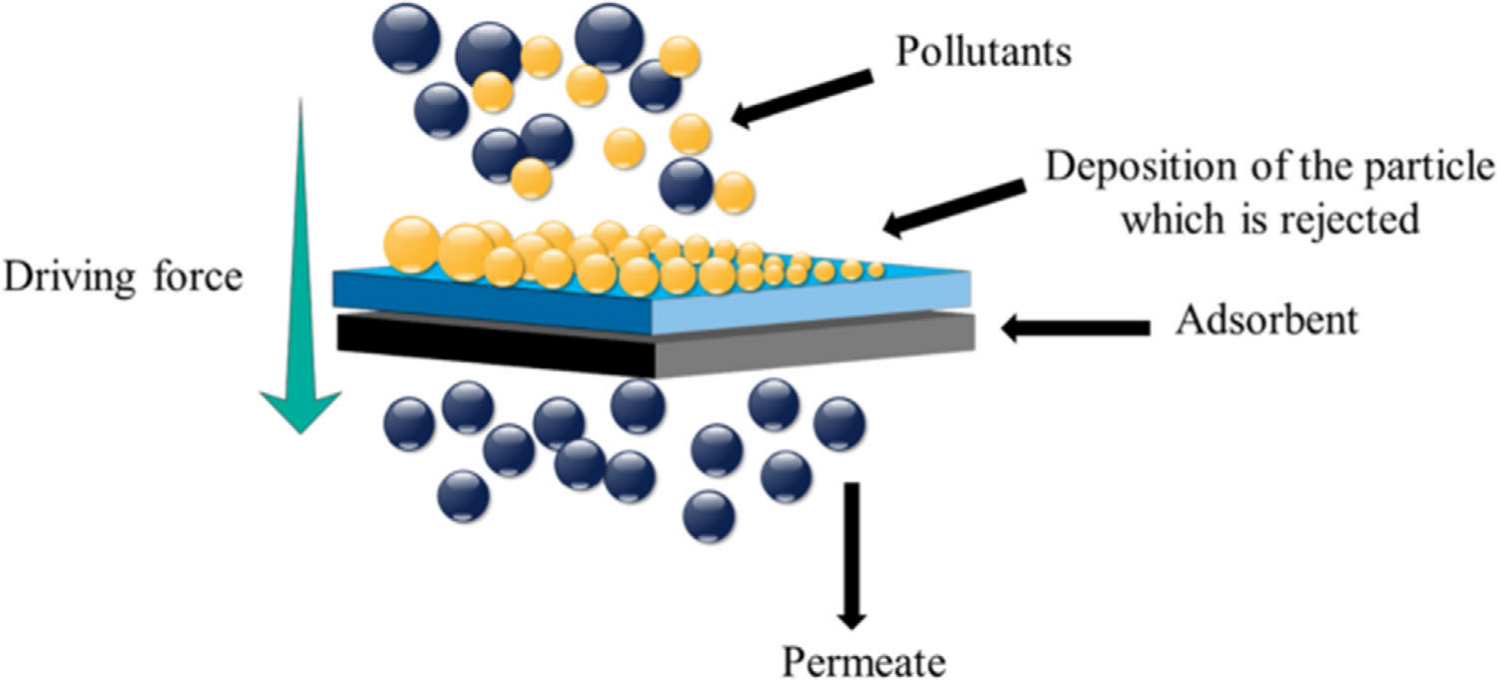
Fundamental adsorption method for the elimination of pollutants onto chalcogenide materials.

Adsorption is a commonly used method for cleaning water of contaminants from aqueous media. Adsorbent can be formulated by doping semiconductor nanomaterials onto a layer of chalcogenide materials.^[^ ^59^, ^169^, ^170^ ^]^ As a result, the contaminants are able to attach to the heterojunctions in a very effective way thereby giving the materials a sorption physiognomy. The process of obtaining the target permeated by interacting contaminated water with a sorbent is shown in **Figure** **11**. The sorption efficiency highlights the sorption sites that are accessible, leading to an improvement in sorption performance (**Table** **3**).^[^ ^43^ ^]^ The contaminant molecules in the bulk media adhere to the surface of the sorbent by physisorption or chemisorption. The distinct forces exerted on particles by the sorbent and bulk media affect the sorption process. The imbalanced forces occurring on the surfaces of the sorbents attract sorbate molecules to them. The superior physical characteristics, including elevated porosity and extensive surface area, render chalcogenides viable sorbents.

**Table 3:** Performance of chalcogenide adsorbents for the removal of organic pollutants from water/wastewater.

Pollutants	Chalcogenides	Initial condition	Removal efficiency [%]	*q*_max_ [mg g^−1^]	Kinetics	Model	Ref.
Congo red	Co_4_S_3_	*t* = 120 min, *C* = 50 mg L^−1^	99.8	3270	PSO	Langmuir	[187]
Congo red	MoS_2_ nanosheet powder		≈95.2 in 20 mins				[188]
EB	MoS_2_ nanosheet powder	*C* = ≈20 mg L^−1^	≈97.1 in 20 mins				[188]
MB	Co_4_S_3_	*C* = 50 mg L^−1^, *t* = 120 mins	97	629	PSO	Langmuir	[187]
MB	MoS_2_/CuS NCs		100	432.68			[189]
MB	MoS_2_		58		PFO		[190]
MB	Ag‐MoS_2_		94		PFO		[190]
MB	Co‐MoS_2_		80		PFO		[190]
MB	Bi‐MoS_2_		72		PFO		[190]
MB	Zr‐MoS_2_		92		PFO		[190]
MB dye	Fe_3_S_4_ hollow sphere	C = 100 mg L^−1^		29.3			[187]
MO	MoS_2_/CuS NCs		48.9	98.78			[189]
MO	Cu_2_FeSnS_4_ hollow chain microsphere	adsorbent dosage of 1 g L^−1^, *t* = 40 mins *C *= 30 mg L^−1^, natural pH, and 298 K	90	123.6 mg g^−1^	PSO	Freundlich	[191]
RhB	Co_4_S_3_		‐	1138	PSO	Langmuir	[187]
RhB	MoS_2_/CuS NCs	*C* = 80 mg L^−1^	93.8 in 30 mins	276.24	PSO	Langmuir	[189]
RhB 6G	MoS_2_/CuS NCs		84.73	211.18			[189]
RhB dye	Cu_2_MoS_4_/g‐C_3_N_4_	pH = 3, 5, and 7, *C* = 30 mg L^−1^	97.5 in 20 mins	420.2	PSO	Langmuir	[177]

Doping chalcogenides with other super materials often enhances their efficiency. This is evident in the research conducted by Liu group where molybdenum disulfide sheet was decorated to enhance pebax‐molybdenum disulfide mixed matrix membranes for environmental remediation application.^[^ ^171^ ^]^ In another instance, Li et al. produced designed a CoMoS_4_ nanostructure for the rapid removal of methylene blue reaching equilibrium within 90 s. This exceptional performance was attributable to the “negative zeta potential” present in chalcogenide, thereby significantly facilitating the mass transfer. The sorption capacity of 292.4 mg g^−1^ obtained was far exceeded that of the previous documented sorption system.^[^ ^172^ ^]^

Several selenide and telluride‐based materials have previously investigated as potential sorbents for the purpose of water purification, including CdTe, CoSe,  MoSe_2_ nanosphere, MoSe_2_^[^ ^59^, ^173^, ^174^, ^175^, ^176^ ^]^ were previously utilized for removing RhB from aqueous environment translating to a maximum sorption capacity = 133 mg g^−1^ in 5 min, whereas activated carbon counterpart necessitated 20 min to reach the equivalent sorption capacity.^[^ ^174^ ^]^ The process of removal of contaminants from a complex matrix is a critical aspect to assess when determining the efficacy of the sorbent materials in water/wastewater treatment.

**Figure 12:**
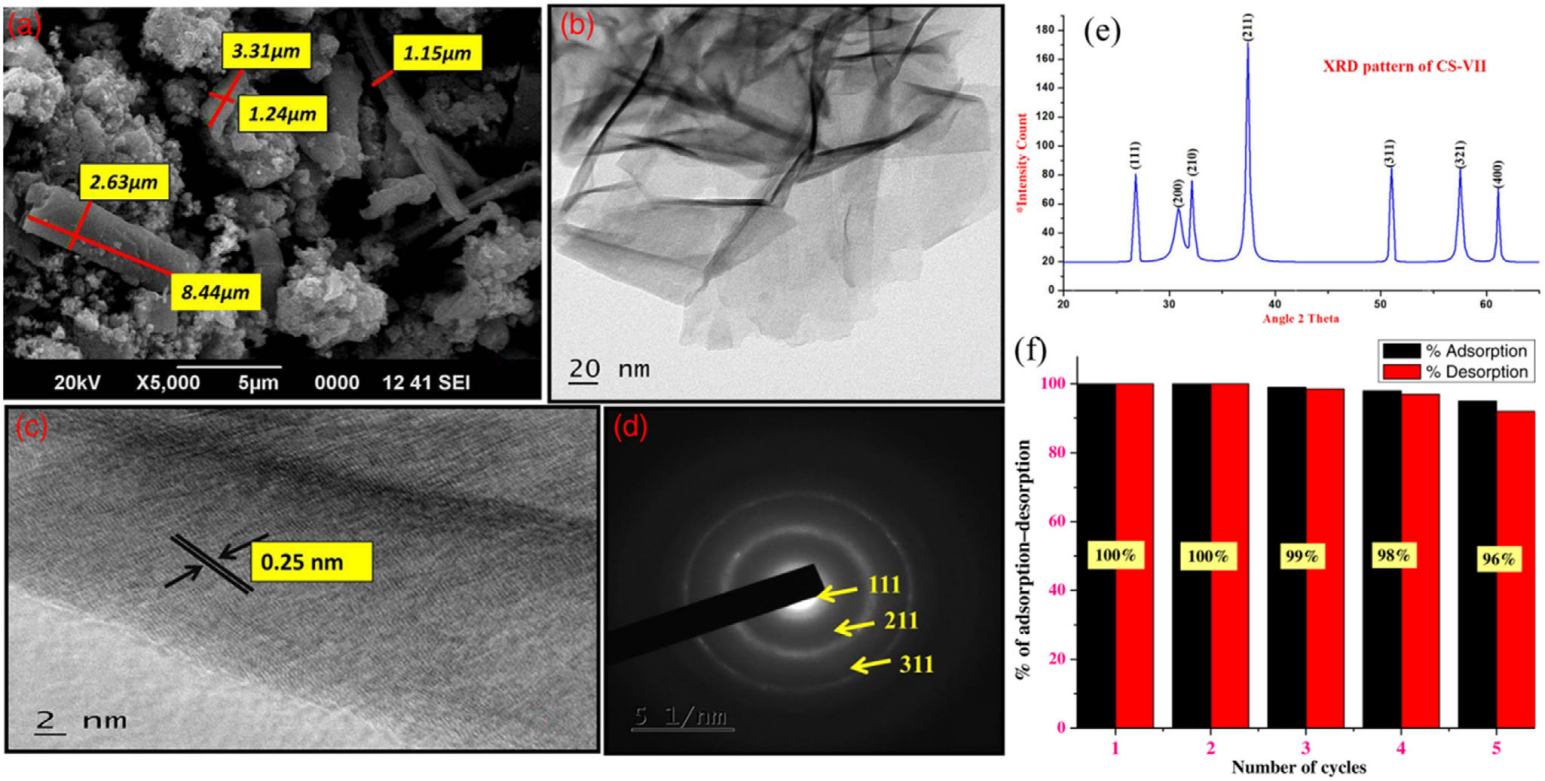
“Nano‐structured morphology of CoSe_2_ nanoflakes viewed using a) FE‐SEM, b) TEM, c) HR‐TEM and d) SAED pattern. e) Typical XRD diffraction pattern of CoSe_2_ nanoflakes which were synthesized using 12 mol L^−1^ KOH matching JCPDS No. 88–1712. f) Percentage change in adsorption with increasing numbers of adsorption and desorption cycles (five) demonstrates the reusability of CoSe_2_ nanoflakes for adsorption of RhoB dye.” Reproduced with permission.^[^ ^175^ ^]^ Copyright 2018, Wiley.

Given that “adsorption” is fundamentally a “surface phenomenon”, the contest for surface areas that are accessible plays a crucial role when addressing water purification issues (Table 3). In their research, Ghaedi and Jah established the effective sorptive capacity of CdTe nanoparticle incorporated into activated carbon for removing sunset yellow dye from actual effluent sample that included a range of other dyes.^[^ ^173^ ^]^ Notwithstanding the existence of alternative dyes, this sorbent demonstrated an impressive percentage removal of 97% in the elimination of sunset yellow dye. It is frequently observed that a substantial sorption capacity is associated with a vast surface area. Nonetheless, additional factors including morphology and surface chemistry also play a crucial role in determining the sorptive capacity of sorbents. Based on this, Asymmetrical, smooth CoSe_2_ nanoflake has been fabricated (**Figure** **12**a–e) through the optimization of KOH solution concentration, facilitated by polyethylene glycol‐400, utilizing an efficient microwave‐assisted technique. The RhB dye removal onto CoSe_2_ has been characterized to possess a relatively modest surface area of 13.2 m^2^ g^−1^.^[^ ^175^ ^]^ This sorbent demonstrated a remarkably elevated sorption capacity of 178.5 g mg g^−1^ which was attributed to the layered morphology of the CoSe_2_ nanoflake. The investigation about the adsorption–desorption dynamics of RhB dye reveals that the as‐obtained successfully utilized for five cycles (Figure 12f). Following the successful execution of two adsorption–desorption cycles, the adsorbent's adsorption capacity only reduced by 1% in the third cycle, yet only experienced a reduction of up to 4% after the fifth cycle.

**Figure 13:**
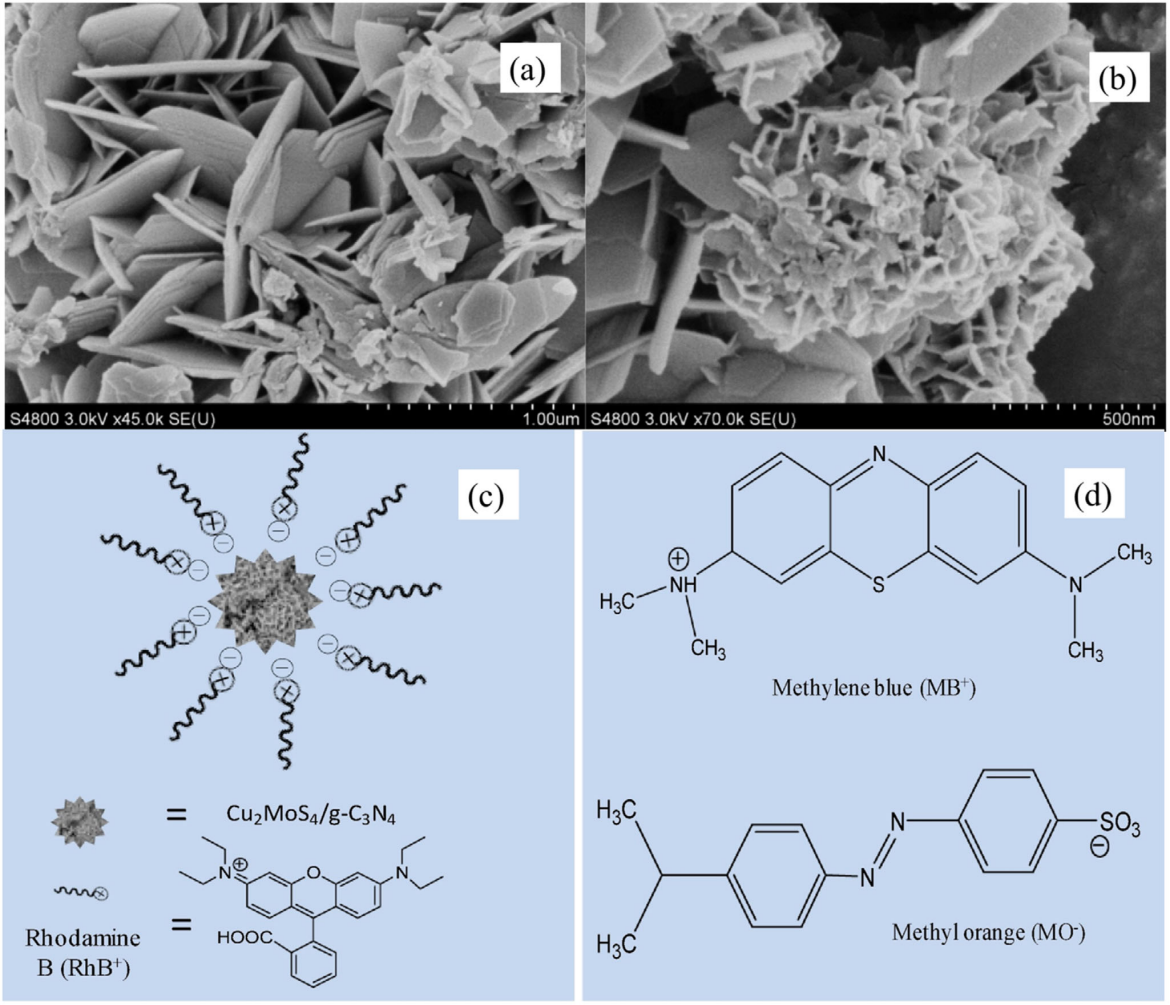
“SEM images of a) Cu_2_MoS_4_‐4/g‐C_3_N_4_ b) Cu_2_MoS_4_‐2/g‐C_3_N. Sorption mechanism of c) Cu_2_MoS_4_/g‐C_3_N_4_ and d) the structure of MB and MO.” Reproduced with permission.^[^ ^177^ ^]^ Copyright 2019, Elsevier.

The composite of g‐C_3_N_4_ and Cu_2_MoS_4_ nanosheets was synthesized through a hydrothermal method and utilized for the RhB removal (**Figure** **13**).^[^ ^177^ ^]^ The *Q*_max_ value for the synthesized Cu_2_MoS_4_/g‐C_3_N_4_, derived from the Langmuir model and fitting the sorption process to a pseudo‐second‐order kinetic model, reached an impressive 420.2 mg g^−1^, achieved within a 20‐minute equilibrium period. The Cu_2_MoS_4_/g‐C_3_N_4_ composite demonstrated the best adsorption within a pH range of 3.5 to 7, whereas adsorption efficiency diminished at a high pH of 9. This phenomenon can be attributed to the enhanced protonation of nitrogen group at elevated pH level and dissociation of carboxylic acid group in RhB. Consequently, the electrostatic repulsion arising from the negatively charged flower‐like Cu_2_MoS_4_/g‐C_3_N_4_ and RhB intensified, resulting in a diminished sorption capacity. The study further elucidated the sorption characteristics of the composite for MO and MB removal, revealing a superior sorption capacity for the positively charged MB, achieving a removal rate exceeding 97.5%, in contrast to the negatively charged MO (Figure 13c,d).

The MoSe_2_ nanospheres, resembling flowers and averaging 200 nm in size, were synthesized through a straightforward solvent‐thermal technique, consisting of ultrathin nanosheet.^[^ ^174^ ^]^ AFM analysis revealed that the thickness of as‐synthesized nanosheet varied from 3.4 to 6.4 nm through the control of the *n*‐alcohol solvent chains. The MoSe_2_ nanosheet synthesized in ethanol solvent demonstrated optimal sorption capability when compared to those produced in other *n*‐alcohol solvents with longer chain carbons. This was attributed to the superior crystallinity and enhanced wettability of MoSe_2_ synthesized in ethanol, compared to other samples. The utmost sorption capability of RhB onto MoSe_2_ nanosheet attained 133 mg g^−1^ within 5 min, whereas the equivalent performance of activated carbon required 20 min.

**Figure 14:**
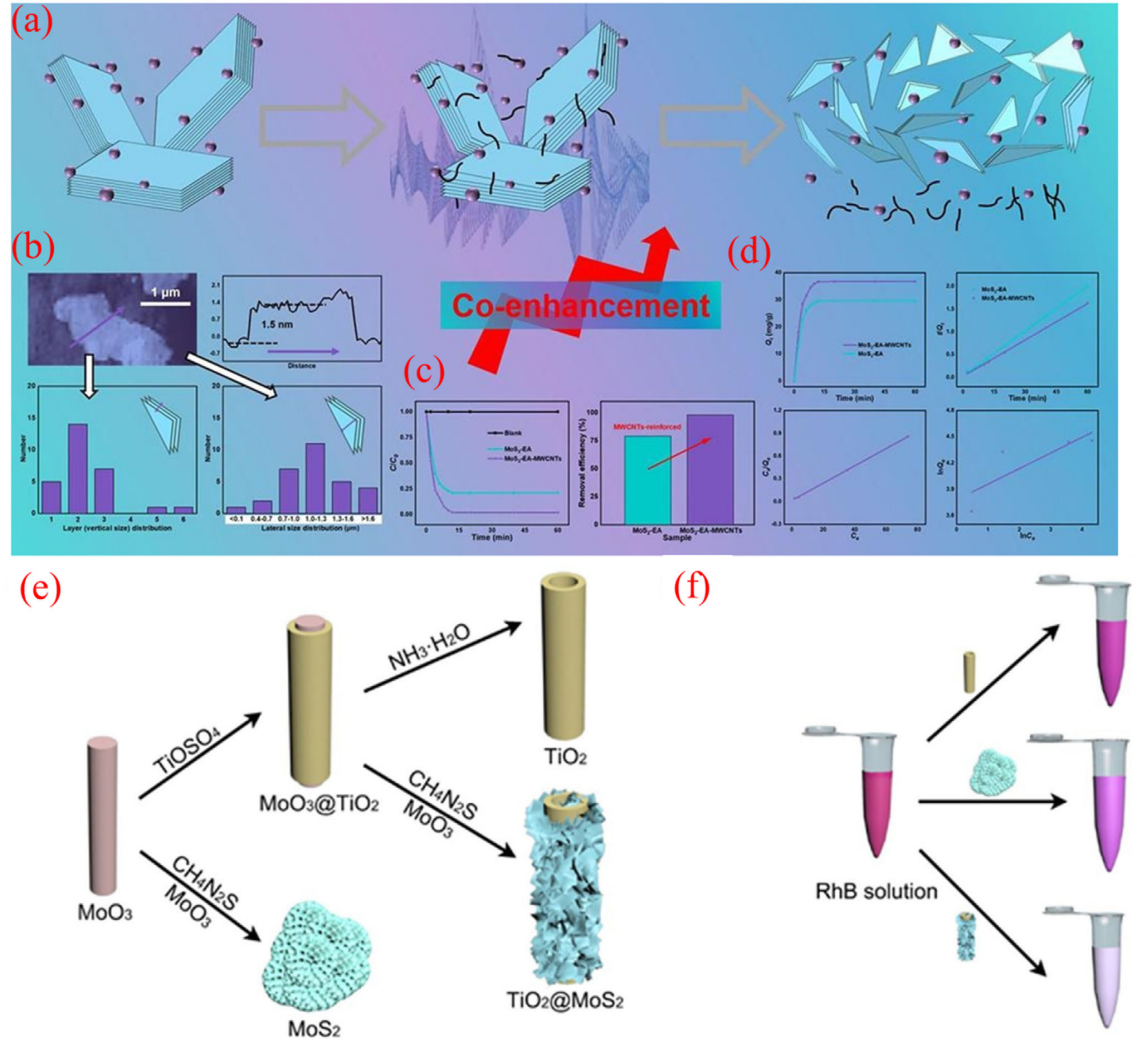
a) Schematic preparation route of MoS_2_‐EA‐MWCNTs. b) AFM image, matching line profile of thickness obtained along the blue line of a singular nanostructured MoS_2_ from MoS_2_‐EA‐MWCNTs, and crystal size distribution for vertical and lateral orientations of MoS_2_‐EA‐MWCNTs. c) sorption activity and removal efficient, d) sorption kinetics and isotherms.^[^ ^178^ ^]^ e) Methodology for synthesizing MoS_2_ nanosheet, TiO_2_ microtube, and TiO_2_@MoS_2_ microtube, together with their f) utilization for the elimination of RhB. Reproduced with permission.^[^ ^179^ ^]^ Copyright 2020, Elsevier.

MoS_2_ chalcogenides have garnered significant interest across diverse research domains owing to their remarkable physical and chemical characteristics. Due to its significant abundance of atomic sulfur, it has been effectively utilized in purifying wastewater and contaminated drinking water. Motivated by this, ultrathin MoS_2_ crystals have been modified with multiwall carbon nanotubes (MWCNTs) to improve the sorption of malachite green in wastewater, achieving a *q*_max _= 87.8 mg g^−1^ as presented in **Figure** **14**a–d.^[^ ^178^ ^]^ In another investigation, hierarchical TiO_2_@MoS_2_ microtube was constructed as effective sorbents for the RhB removal (Figure 14e,f).^[^ ^179^ ^]^ The synergistic sorption between mesoporous TiO_2_, MoS_2_ nanosheets, and 1D hierarchical hollow microtube resulted in a composite that exhibited an impressive RhB *q*_max _= 94.01 mg g^−1^, along with specific surface area surpassing those of MoS_2_ and TiO_2_. The remarkable sorption capacity of the functionalized hollow microtube positions them as an advantageous material for removing organic contaminants from water/wastewater. Other similar MoS_2_‐based materials have been prepared for the effective removal of dyes from water/wastewater.^[^ ^180^, ^181^, ^182^, ^183^, ^184^, ^185^, ^186^ ^]^

Li and colleagues presented the design of ternary CoMoS_4_ nanorod, which exhibits a remarkable capacity for the fast MB sorption. The attainment of sorption equilibrium was achieved within 90 s utilizing CoMoS_4_ nanorod. The negative zeta potential of Chalcogenide materials was identified as a contributing factor that facilitated mass transfer. Furthermore, the *q*_max _= 292.4 mg g^−1^ was obtained which surpasses those documented for alternative sorption processes, including pristine graphene, activated charcoal, and molybdenum sulfide. A uniform hollow sphere of Fe_3_S_4_ has been reported for removing MB from a solution of water.^[^ ^48^ ^]^ The as‐prepared colloid sphere demonstrated a *q*_max _= 29.3 mg g^−1^ at a concentration of 100 mg L^−1^ for MB. The employment of alternative Fe‐based complexes, such as a‐FeOOH and a‐Fe_2_O_3_, yielded *q*_max _= 80 mg g^−1^. highlighting the efficacy of the hollow chalcogenides.

**Figure 15:**
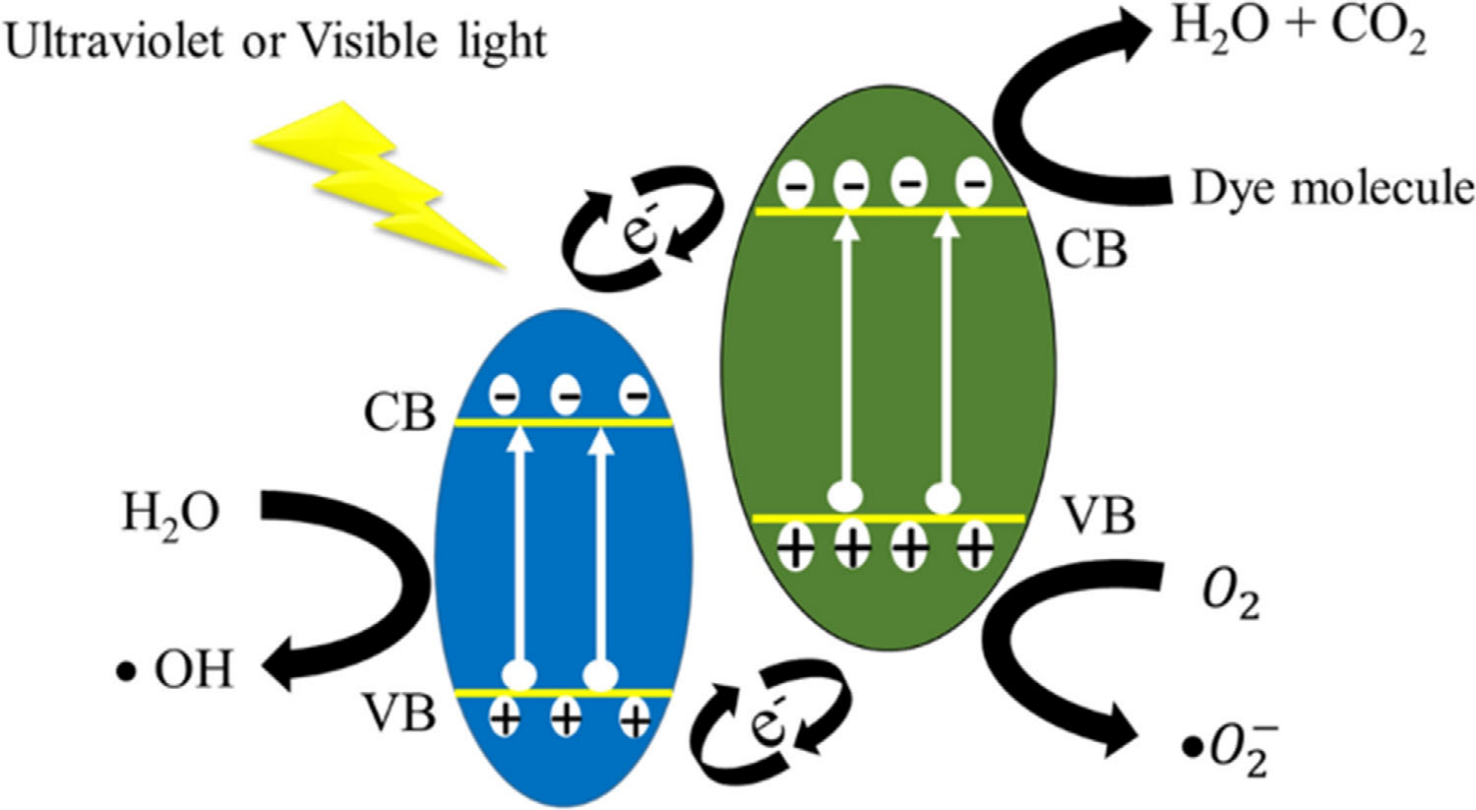
Photodegradation of dyes.

Photocatalysis typically utilizes semiconducting photocatalysts that operate effectively under UV/Vis light, producing electron–hole pairs to target and degrade pollutants.^[^ ^192^ ^]^ In recent years, advancements in photocatalysts have come to light, attributed to the tunable bandgaps which function effectively in the visible region of the UV spectrum.^[^ ^170^ ^]^ Moreover, photocatalysis is attracting significant attention in water/wastewater treatment due to the compatibility of various photocatalysts (**Table** **4**). Similarly, chalcogenide materials have demonstrated outstanding properties in UV/Vis light.^[^ ^16^ ^]^ Additionally, recent investigations on chalcogenide materials have significantly enhanced exploration and innovation in environmental applications thereby finding extensive usage photocatalysis. When UV/vis light strikes the photocatalysts, the valence electron(s) in the valence band gets excited to the conduction bands due to the absorption of the appropriate photon(s) (those with energy exceeding the band gaps). This phenomenon leads to the creation of electron–hole pairs. Additionally, the electron and hole interact with dissolved O_2_ to produce superoxide radical (O_2_^−^), whereas the hole also reacts with H_2_O to create hydroxyl radical (OH). In the course of time, dyes or organic contaminants then undergo degradation into water, CO_2_, and other simple organic molecules. The expected photodegradation is illustrated in **Figure** **15**.

**Table 4:** Performance of chalcogenide materials for the photocatalytic removal of organic pollutants removal from water/wastewater.

Pollutants	Chalcogenides	Light sources	Efficiency	Refs.
2,4‐dichlorophenol	Ag/Ag_2_S/CuS	XL (0.3 KW)	82%, *t *= 240 min	[194]
Atrazine	CuS@rGO	Xe lamp (300 W)	100%, *t* = 50 min	[195]
Atrazine	CuS@rGO	XL (0.3 KW)	100%, *t* = 20 min	[195]
Benzophenone‐1 (Ph_2_CO).	Cu_2_WS_4_/BiOCl	XL (250 W)	99%, *t* = 40 min	[196]
Bisphenol A	Citrate‐modified CuFeS_2_	Fluorescent lamp (4 W)	97%, *t* = 60 min	[197]
Doxycycline	AgInS_2_─TiO_2_	Hg vapor light (0.125 KW)	95%, *t *= 180 min	[198]
Lomefloxacin	Cu_2_WS_4_	LED irradiation (20 W)	60%, *t* = 120 min.	[196]
MB	Cu_2_ZSnS_4_	Visible light,	60%, *t *= 90 min	[199]
MB	Cu_2_FeSnS_4_	Sunlight	81%	[176]
MG	CuCo_2_S_4_/RGO NC (@3% rGO loading)	Tungsten Halogen lamp (0.5 KW)	92%, *t *= 360 min	[200]
MO	Sb_2_S_3_	Sunlight	47%	[201]
MO	Cu_2_SnS_3_/rGO	XL (0.3 KW)	94%	[202]
MO	Ag_2_S/AgInS_2_	Visible light	80%	[203]
MO	MoS_2_/ZnIn_2_S_4_	XL (0.3 KW)	84%	[204]
MO, RhB, and paracetamol	ZnIn_2_S_4_/MoO_3_	Halogen lamp (0.5 KW)	98%, *t *= 80 min.	[205]
Norfloxacin	CdS/Au/TiO_2_	XL (0.035 KW)	65%, *t *= 60 min	[203]
RhB	Cu_2_WS_4_/NiTiO_3_	XL (300 W)	98%, *t *= 20 min	[206]
RhB	BaAu_2_S_2_	XL (1 KW), 420 filter, *λ* = 420 nm	45%, *t* = 240 min	[207]
RhB	Cu_2_SnS_3_/rGO	XL (0.3 KW)	96%	[202]
RhB	CdS@MoS_2_ core@shell	XL (0.3 KW)	80%	[208]
RhB dye	CdS/EU‐12	Halogen (200 W) lamp, stirring speed = 800 rpm, pH = 3.	98.6, *t *= 180 min	[208]
Tartrazine dye	CuFeS_2_	FL (85 W)	99.1%, *t* = 40 min	[197]
TC HCl	Cu_2_WS_4_/NiTiO_3_	XL (300 W)	89%, *t* = 60 min	[206]
Thiophene	MoS_2_@rGO	Hg visible light/125 W	100%, *t* = 75 min	[209]
Thiophene	CdSe/rGO	Hg lamp (125 W)	100%, *t* = 90 min	[210]

Xenon lamp: XL.FL: Fluorescence lamp.

Ramalingam et al. doped the Ni^+^‐Co^2+^‐modified MnO_2_ denoted as Ni‐Co‐MnO_2_ with MoS_2_ and applied the as‐obtained material for sonocatalytic dye degradation.^[^ ^193^ ^]^ It was observed that at 10% and 5% MoS_2_ loading onto Ni─Co─MnO_2_ denoted as MoS_2_/Ni─Co─MnO_2_, a complete discoloration of Congo red(CR) dye was achieved, alongside improved sorption activity due to boosted surface areas compared to undoped MnO_2_. The degradation cycle's stability and reusability exhibited effective consistency of up to 95%.

### 4.2 Pharmaceutical and Personal Care Products

The efficacy of traditional wastewater treatment strategies in eliminating PPCPs from water/wastewater has been notably inadequate. In a study conducted by He and colleagues,^[^ ^211^ ^]^ polypyrrole was functionalized with Cu_2_MoS_4_ magnetic composite (Cu_2_MoS_4_‐Fe_3_O_4_@PPy) was produced and employed for sorptive removal of PPCPs. The as‐obtained composite revealed synergistic outcomes and displayed remarkable adsorption capabilities for ketoprofen and indomethacin, achieving efficiencies of 88.6% and 97.1%, respectively, at 10 mg L^−1^ initial concentration across a broad pH spectrum of 5 to 11. The Cu_2_MoS_4_‐Fe_3_O_4_@PPy demonstrated facile magnetic separation and exhibited the capability for reuse across eight cycles of adsorption‐desorption, achieving an impressive removal efficiency of 83%. Following the deposition of silver nanoparticles on the composite material (Cu_2_MoS_4_‐Fe_3_O_4_@PPy), the resulting Cu_2_MoS_4_‐Fe_3_O_4_@PPy.Ag composites were subsequently utilized in the photodegradation of MG dye under visible light, achieving a remarkable removal efficiency of 94.8% within one hour. The Cu_2_MoS_4_‐Fe_3_O_4_@PPy.Ag exhibited enhanced selectivity for MG in both dual and ternary dye solutions.

**Figure 16:**
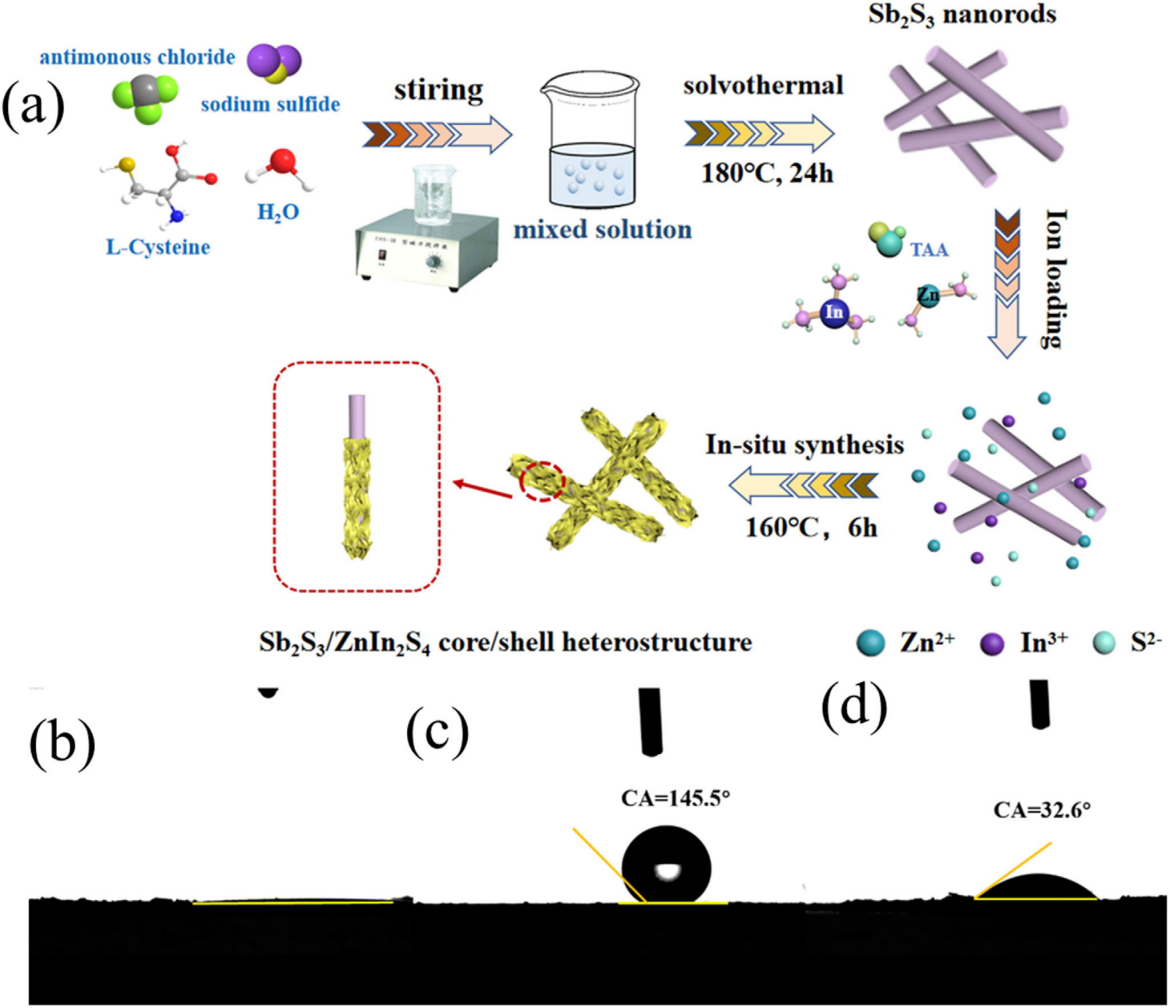
a) Schematic representation for the fabrication of a ZnIn_2_S_4_ modified Sb_2_S_3_ hybrid heterostructure. Water contact angles of b) ZnIn_2_S_4_; c) Sb_2_S_3_; and d) SB‐ZIS‐2. Reporduced with permission.^[^ ^215^ ^]^ Copyright 2017, Elsevier.

The elevated mobility carrier and exceptional chemical stability of conducting polymer polypyrrole (PPy) were recently combined with ZnIn_2_S_4_ for the effective photodegradation of the antibiotic chloramphenicol.^[^ ^212^ ^]^ The PPy‐ZnIn_2_S_4_ combination decomposed CHL with a mineralization rate of 48.5%, which is double that of pristine ZnIn_2_S_4_. The CHL degradation involved multiple stages, including the breaking of C─OH, phenylnitryl C─N , and C─Cl bonds.^[^ ^213^ ^]^ Recently, a new hierarchical 1D/2D core‐shell Sb_2_S_3_‐ZnIn_2_S_4_ (SB‐ZIS) heterostructures exhibiting excellent photocatalytic efficiency for organic pollutant remediation was devised and synthesized using a straightforward one‐step hydrothermal technique (Figure 14a).^[^ ^214^ ^]^ The synthesized SB‐ZIS heterostructure, characterized by the uniform growth of ZnIn_2_S_4_ nanosheets on Sb_2_S_3_ nanorods, established a compact and extensive interface that enhanced light absorption, increased surface area, reduced electrical transmission distances, and facilitated the separation and movement of photogenerated carrier. The authors endeavored to effectively break down tetracycline hydrochloride utilizing a Sb_2_S_3_‐ZnIn_2_S_4_ core–shell structure. The establishment of close interaction between Sb_2_S_3_ nanorod and ZnIn_2_S_4_ sheet resulted in a maximum removal rate of 0.514 per hour due to reduced charge carrier transport distance and enhanced surface area. The decomposition process (**Figure** **16**f) of TCH involves electron(s) on the surface of the hybrid composites reacting with oxygen to generate ·O_2−_, that serves as the primary active species in the degradation of TCH. Simultaneously, the holes exerted a direct influence on the TCH organic molecule.^[^ ^214^ ^]^

Another study^[^ ^213^ ^]^ has analyzed several intermediates produced and delineate the conversion pathway of photodegrading five distinct antibiotics including tetracycline hydrochloride, erythromycin, chloramphenicol, rifampicin and linomycin hydrochloride) utilizing ZnIn_2_S_4_ in visible light irradiation. The conversion routes associated through the synthesis of many intermediates were examined utilizing “electron paramagnetic resonance analysis and liquid chromatography‐mass spectrometry‐ion trap‐time of flight (LCMS‐IT‐TOF)” techniques. The rifampicin degradation involves the detachment of the nitrogen‐containing ring through the breakage of C─N bond, N─N bond, and the demethylation of the methoxyl and acetoxyl groups. The proposed conversion paths for degrading erythromycin involve the cladinose and desosamine sugar removal, succeeded by processes of deethylation and demethylation. Degrading chloramphenicol encompasses the breaking down of dichloroacetamide, decarbonylation, the methanol removal, and the cleavage of the benzene ring. The pathway for degrading linomycin hydrochloride involves the detaching methylthio, amide and pyrrolylene groups, as well as processes such as hydroxylation, demethylation, and the pyranose ring cleavage. The photodegradation of tetracycline HCl involved the detaching N‐dimethyl, primary amine, and amide groups, alongside ring‐opening reactions.^[^ ^213^ ^]^

**Figure 17:**
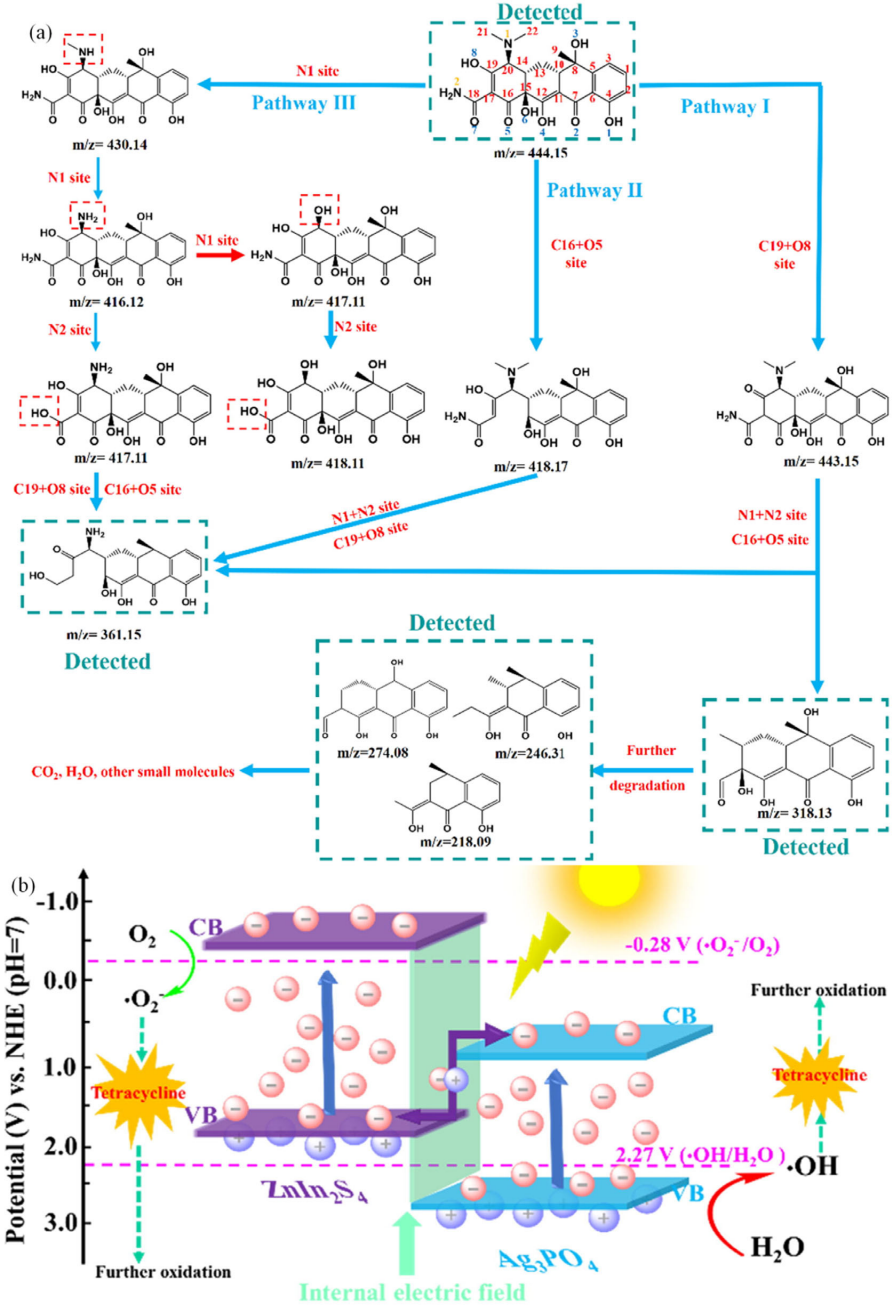
a) Proposed route and intermediate products for degradation of tetracycline. b) Mechanism for the degradation of tetracycline. Reproduced with permission.^[^ ^217^ ^]^ Copyright 2021, Elsevier.

An endeavor to improve the absorption of visible light in BiVO_4_ was undertaken through its coupling and ZnIn_2_S_4_ and g‐C_3_N_4_.^[^ ^216^ ^]^ The ternary ZnIn_2_S_4_‐g‐C_3_N_4_/BiVO_4_ demonstrated outstanding performance in the visible light photodecomposition of the metronidazole antibiotic, achieving a degradation efficiency of 59% and rate constant = 0.00681 per minute. The potential photodegradation route was examined through three conceivable Z‐scheme approaches. The enhanced surface area, effective absorption of visible light by the composite, and the Z‐scheme degradation pathway contribute significantly to its outstanding functionality.^[^ ^216^ ^]^ In another study, a novel photodegradation system for the elimination of tetracycline employing a hierarchical Ag_3_PO_4_@ZnIn_2_S heterostructure has been reported.^[^ ^217^ ^]^ It was demonstrated that decorating ZnIn_2_S_4_ with Ag_3_PO_4_ nanoparticles achieved an impressive efficiency of 92.3%. The proposed degradation pathway posits that electron transport plays a crucial role in maintaining the stability of Ag_3_PO_4_ against photo corrosion (**Figure** **17**).^[^ ^217^ ^]^

**Figure 18:**
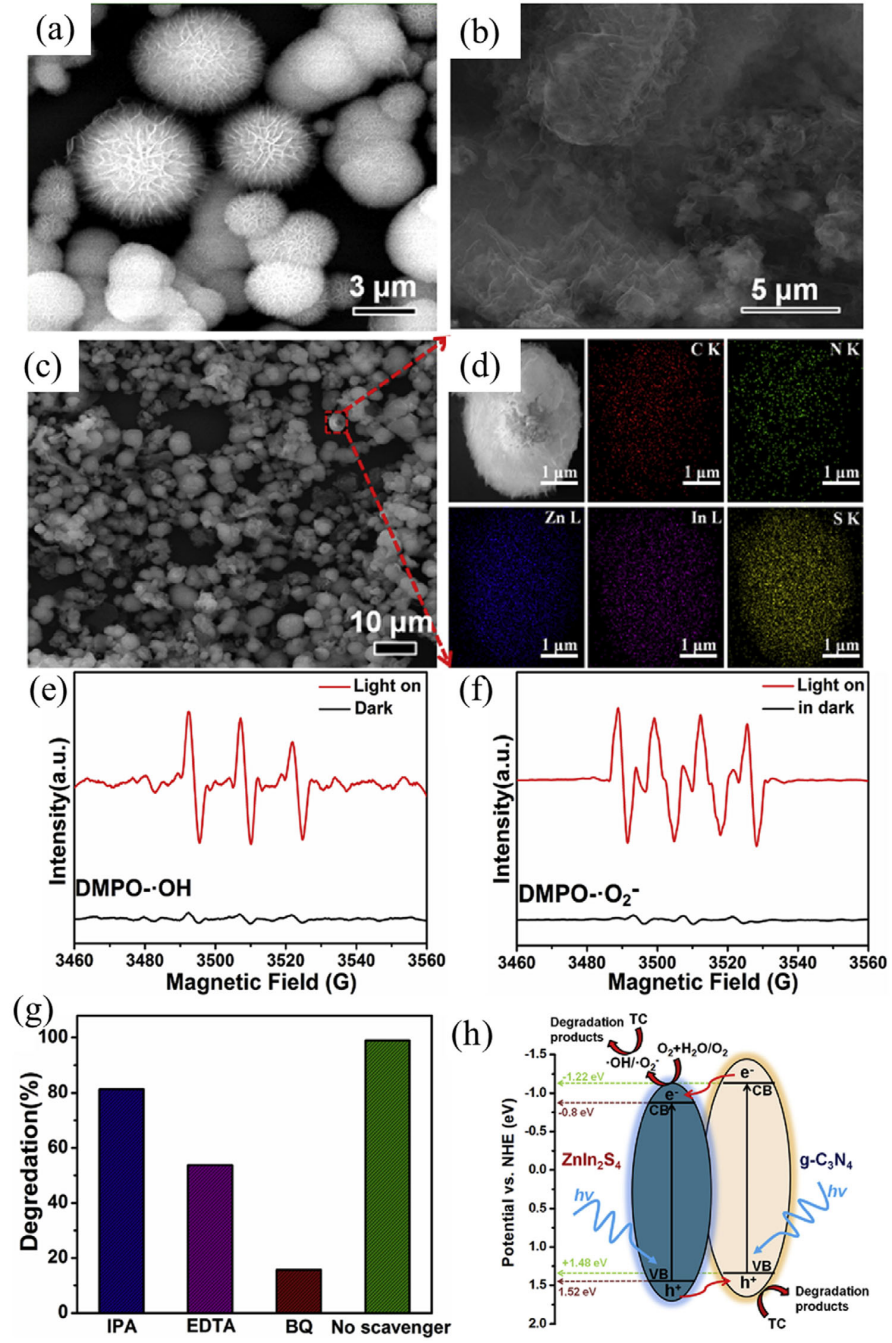
“SEM images of a) ZnIn_2_S_4_, b) g‐C_3_N_4_, c) 50% CZ, d) corresponding mapping images. ESR spectra of 50% CZ in dark and under visible light (*λ* > 420 nm): e) DMPO‐^•^OH in aqueous dispersions and f) DMPO‐^•^O_2_^−^ in methanol dispersions. g) Trapping experiment of active species during the photocatalytic breakdown of tetracycline over 50% CZ under visible light irradiation. h) The potential mechanism behind the improved photocatalytic activity of the g‐C_3_N_4_/ZnIn_2_S_4_ heterojunction.” Reproduced with permission.^[^ ^215^ ^]^ Copyright 2017, Elsevier.

Furthermore, the efficacy of the g‐C_3_N_4_/ZnIn_2_S_4_ composites in facilitating the photodegradation of tetracycline (TC), a prevalent antibiotic utilized in veterinary applications, thereby contributing to the mitigation of antibiotic pollution in aquatic environments (**Figure** **18**).^[^ ^215^ ^]^ The heterojunction formed in g‐C_3_N_4_/ZnIn_2_S_4_ was characterized by appropriately aligned band edge position, thereby demonstrating a TC degradation when subjected to simulated sunlight. Due to the band edge potential of ZnIn_2_S_4_ and g‐C_3_N_4_, electron from the conduction band of g‐C_3_N_4_ transition to the conduction band of ZnIn_2_S_4_, while the photogenerated hole move from the valence band of ZnIn_2_S_4_ to the valence band of g‐C_3_N_4_, thereby facilitating the separation of charge carriers. The conduction band potential of ZnIn_2_S_4_ exhibits a more negative value compared to the reduction potential of molecular oxygen (O_2_/^•^O_2_^−^) (Figure 18h). Consequently, ^•^O_2_^−^ is produced at the conduction band of ZnIn_2_S_4_, accompanied by the generation of the OH^•^ radical through the partial ^•^O_2_^−^ conversion. The vacancies present in the valence band of g‐C_3_N_4_ facilitate the oxidation of the pollutants.^[^ ^215^ ^]^

### 4.3 Pesticides

**Figure 19:**
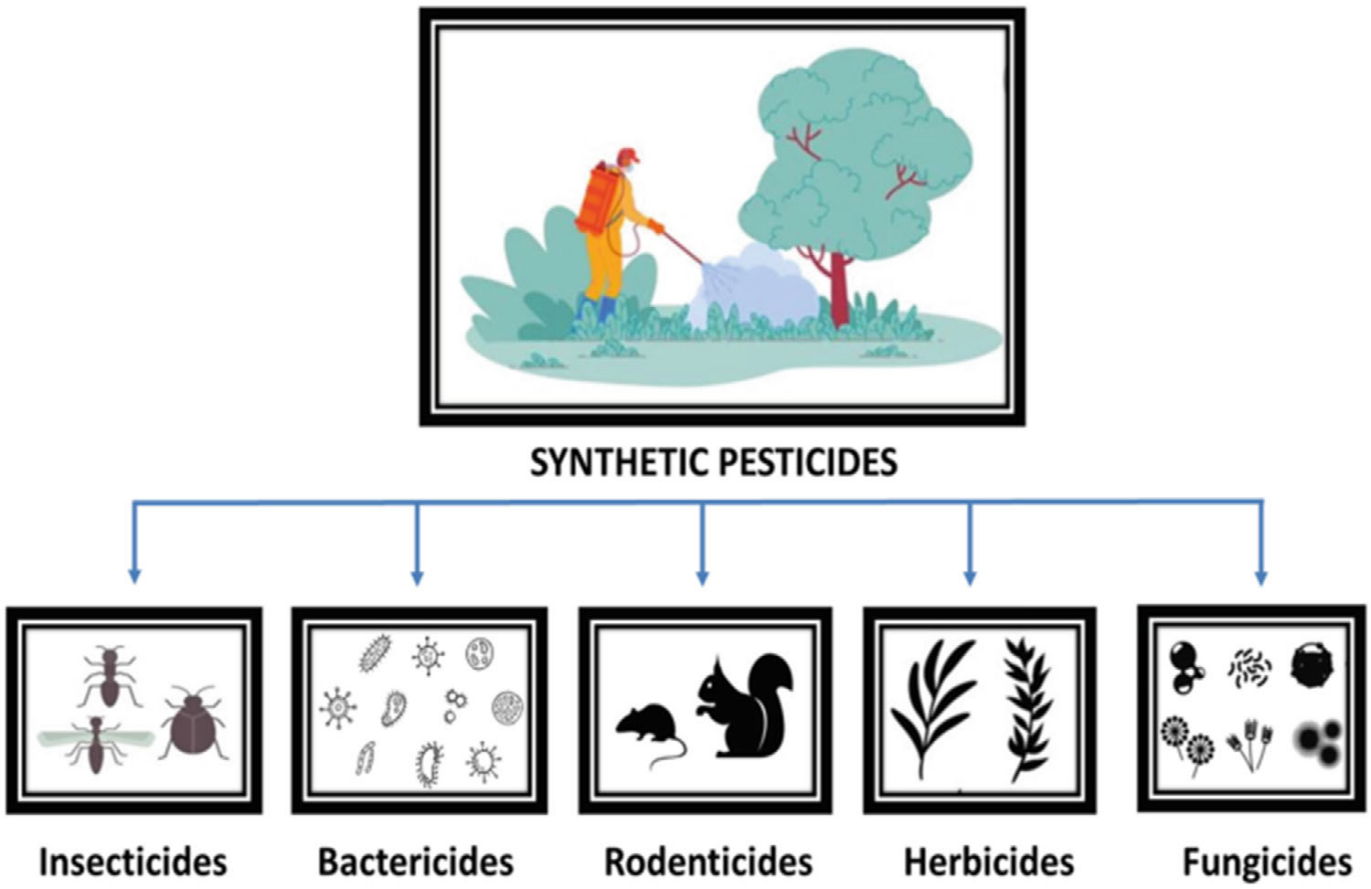
Classes of synthetic insecticides according to pest kinds[218] 2022, Springer

Pesticides have become widely recognized for their distinctive toxicological and chemical characteristics. Consequently, they persist as components of the ecosystem to enhance agricultural production. Furthermore, it facilitates sustainable food production and diminishes pest‐related disturbances. They are synthesized chemicals that incapacitate or immediately exterminate the pests. They are very effective and rapidly efficient in managing targeted populations of pests. Chemical pesticides provide longer‐lasting control and extended residual action in field circumstances.^[^ ^218^ ^]^ They may be categorized according to the kinds of pest(s) they eliminate (**Figure** **19**) and based on their chemical structures. Pesticides are also categorized according to the sorts of pests they eliminate. For instance, insecticides eliminate insects, bactericides eradicate bacteria, herbicides destroy weeds, fungicides combat fungus, and rodenticides exterminate rats.^[^ ^219^ ^]^

Research indicates that just 0.1% of the whole pesticides effectively reach their intended target organism, leaving 99.9% entering the environment.^[^ ^220^, ^221^ ^]^ Despite proper applications, several chemicals infiltrate the ecosystem via water runoff, resulting in the pollution of water surface, estuaries, groundwater, marine ecosystems, and soil deposits, frequently enduring for extended durations. An assessment of arable soil in Europe, conducted months post‐application, identified pesticide levels above danger thresholds for locations with known endocrine disruptors and carcinogens.^[^ ^222^ ^]^ These substances are subsequently dissolved by biological, photolytic, or chemical processes; however, if not decomposed, they remain in the water cycle or are absorbed by other species, therefore infiltrating the food chain.^[^ ^219^ ^]^ Certain chemicals exhibit higher persistence owing to an inherent structural resilience of their compounds against environmental degradation or abiotic factors. For example, organochloride insecticides, including DDT and derivatives, possess half‐lives between 2 and 15 years.^[^ ^223^ ^]^ Persistent chemicals evaporate and condense in the water cycle, traversing considerable distances.^[^ ^224^ ^]^ The longer‐range atmospheric transportation of unrelenting organic pesticides demonstrates that pesticide contaminations are not confined to particular nations or areas; the pollutants and their accompanying damage are disseminated by geochemical process.^[^ ^225^ ^]^

Pesticides and herbicides are by‐products that are challenging to degrade due to their intrinsic biological and chemical stability. The previous report shows that effectively engineered heterojunctions involving g‐C_3_N_4_ and ZnIn_2_S_4_ for photodegradation of 2,4‐dichlorophenoxyacetic acid (2,4‐D), a key constituent utilized in herbicide, could be regarded as promising approach. The as‐0btained ZnIn_2_S_4_/g‐C_3_N_4_ composite exhibited superior photocatalytic efficacy due to improved charge carrier separations thereby exhibiting a five cycle photostability, mostly due to the active species ^•^O_2_− and h+.^[^ ^226^, ^227^ ^]^ A comparable effort was undertaken with the ZnIn_2_S_4_/g‐C_3_N_4_ composites to examine the photodegradation of phenol.^[^ ^228^ ^]^ Interestingly, the ZnIn_2_S_4_/g‐C_3_N_4_ composites, having an optimum doping of 40% g‐C_3_N_4_ using hydrothermal process, demonstrated exceptional photo‐activity phenol degradation within 4 h of visible light irradiation. The improved photoactivity is ascribed to the compatible band structures and close contact surfaces between g‐C_3_N_4_ and ZnIn_2_S_4_. Brominated phenol is a prevalent contaminants in marine environments due to extensive applications as pesticide, wood preservative, and some pharmaceutical intermediate.^[^ ^229^ ^]^ Zerovalent iron nanoparticles combined with ZnIn_2_S_4_ have been successfully employed for 2,4,6‐TBP debromination and mineralization. The debromination route was elucidated using the LCMS approach, which included both reductive and oxidative debromination reactions. Bo et al.^[^ ^230^ ^]^ used Pd‐doped ZnIn_2_S_4_ for eliminating atrazine in direct sun illumination. In photocatalytic process, the electron entering the ZnIn_2_S_4_ conduction band are transferred to vacant bands of Pd^2+^ ions, inhibiting the electron–hole pair recombination, resulting in a photo‐elimination of atrazine achieving a 90% efficiency after 60 min.

Another study reported the development of WO_3_/ZnIn_2_S_4_ Z‐scheme heterojunctions for degrading hazardous nitenpyram chemical in water. This photocatalyst demonstrated a superior elimination rate compared to that of individual heterostructure components, yielding a photodegradation rate constant = 0.042 per min.^[^ ^231^ ^]^

**Figure 20:**
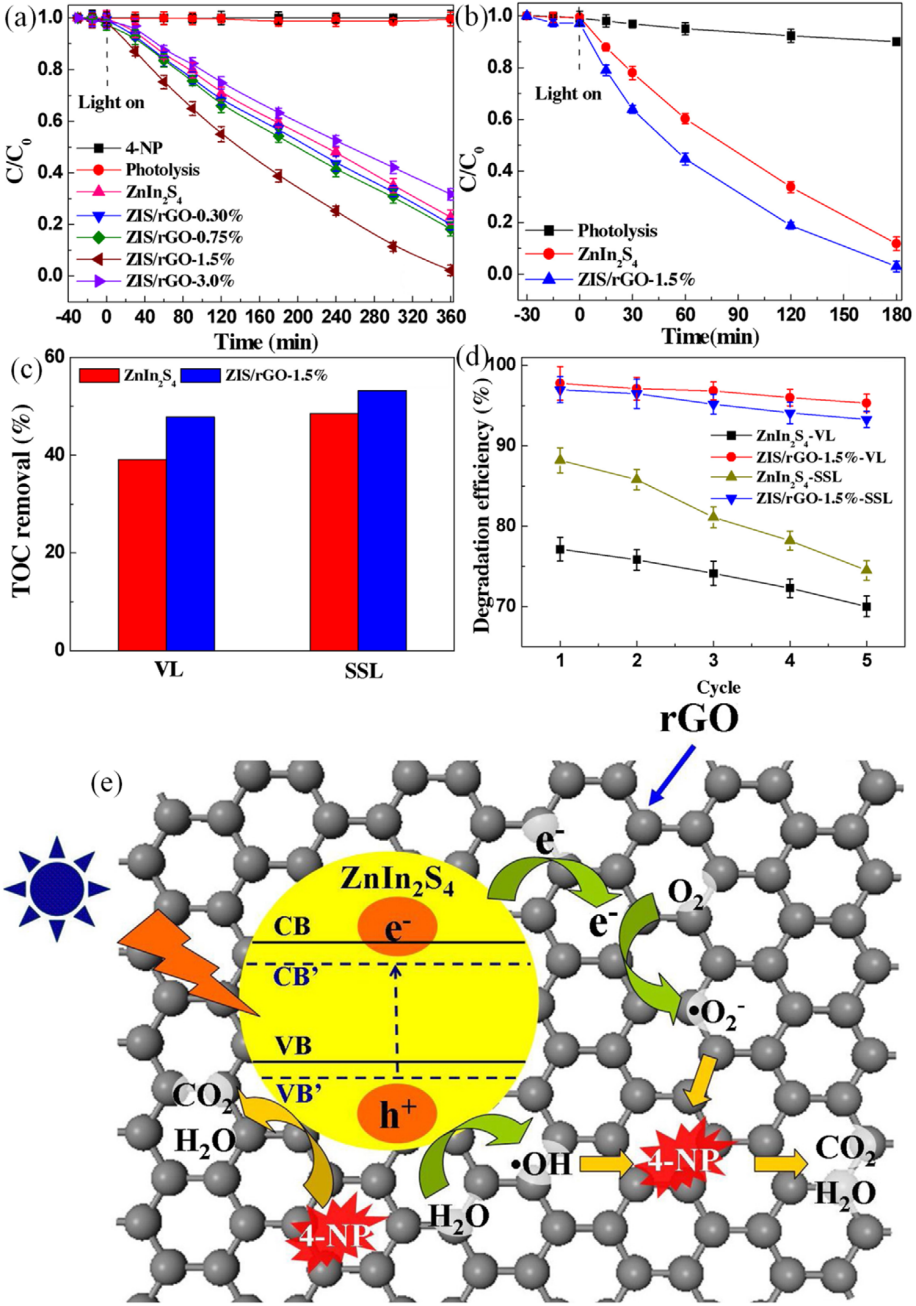
Photoactivity of ZnIn_2_S_4_ and ZnIn_2_S_4_/rGO composites for the breakdown of 4‐NP under visible light (a) and simulated solar light (b) irradiation, c) total organic carbon removal efficiency of 4‐NP onto ZnIn_2_S_4_/rGO–1.5% and ZnIn_2_S_4_, d) photostability assessment of ZnIn_2_S_4_/rGO–1.5% and ZnIn_2_S_4_ for 4‐NP photodegradation. e) Schematic mechanism of 4‐NP photodegradation process. Reproduced with permission.^[^ ^232^ ^]^ Copyright 2015, Elsevier.

Similarly, the cross‐linked and stable ZnIn_2_S_4_/rGO composites have been created for efficient sunlight‐driven remediation of 4‐nitrophenol.^[^ ^232^ ^]^ As presented in **Figure** **20**, the chemical interactions among the rGO sheets and ZnIn_2_S_4_ nanosheets via a Zn─O─C covalent bond enhance photoactivity. It was observed that the covalent bonding enhances the structural integrity and corresponding stabilities of the composites, hence increasing resistance to photo corrosion when exposed to sunlight.

A thin‐layer film containing the ternary metal sulfide Zr_2_S_3_‐BaS‐Cr_2_S_3_ has been produced using physical vapor deposition and subsequently characterized using different analytical techniques.^[^ ^233^ ^]^ The obtained material exhibited a mixture of morphology characterized by the presence of flake and particle resembling the grains of rice. The nanocomposites exhibited an average crystallite size of 21.8 nm, as ascertained via XRD analysis and a band gap energy = 3.7 eV was recorded. Furthermore, the photocatalytic characteristics of the as‐obtained samples for the environmental cleanup of various organic pollutants, such as pesticide zoxamide, phenol, and methylene blue dye. It was observed that the degradation rate decreased to merely 73% following four cycles of photocatalytic processes.

## 5 Comparing Adsorption and Photocatalytic Approaches for Water/Wastewater Treatment

Adsorption and photodegradation are two effective and quick methods for eliminating organic pollutants from water/wastewater. Because photocatalysts are known to be environmentally benign while mitigating the drawbacks associated with adsorption—namely, the transport of contaminants from one phase to another photocatalytic degradation is a sustainable technique. However, the adsorption process generates secondary waste, which raises serious concerns about how to dispose of spent adsorbents.^[^ ^234^, ^235^ ^]^ The organic pollutants are converted or destroyed into less hazardous byproducts or fully mineralized to produce CO_2_ and H_2_O as final results using the photocatalytic remediation technique. This method has an advantage over adsorption since CO_2_ could be retained and utilized as a source of renewable energy, and H_2_O may be further processed to evolve oxygen and hydrogen. By eliminating the organic pollutants entirely, photodegradation has been shown to be more effective than adsorption. Reusable materials are essential for sustainable engineering when it comes to wastewater treatment.

Desorption has been established as a regeneration approach for adsorption systems. Therefore, finding an economical, environmentally acceptable elutant that would not harm the sorbent materials is crucial.^[^ ^236^ ^]^ The desorption effectiveness of acid eluents for regeneration students has been recorded to the highest, when compared with other desorption agents such chelating acids, salts, acids, and alkalis.^[^ ^237^ ^]^ Biological, thermal, and chemical methodologies are other ways to regenerate exhausted sorbents.^[^ ^238^ ^]^ While this is not the case for photocatalysts because the organic molecule has been mineralized, reusing sorbents over multiple cycles may cause undesorbed organic molecules to leach into the solution, thus decreasing the sorbent's efficiency.^[^ ^239^ ^]^ The ability of photocatalysts to regenerate and reuse across many cycles in the breakdown of organic pollutants has been investigated. For example, AgCl/Bi_24_O_31_Cl_10_ as a visible light photocatalyst was shown to demonstrate 79% photodegradation of tetracycline even after three cycles.^[^ ^240^ ^]^

It is interesting to note that adsorption is essential to the majority of photodegradation processes since the pollutants are initially adsorbed on the photocatalyst's surface, where photodegradation occurs via the hole or active species. Since the photocatalyst's surface area is crucial for electron injection, numerous studies have shown that adsorption improves photocatalytic degradation. Therefore, materials possessing photocatalytic and adsorptive capabilities have been developed for several research.^[^ ^241^ ^]^ In addition, it has been shown that adsorption improved the photodegradation of MB over CeO_2_/g‐C_3_N_4_.^[^ ^242^ ^]^

## 6 Challenges and Future Research Priorities

Chalcogenides exhibit a wide array of applications across the fields of engineering, materials science, and medicine.^[^ ^243^, ^244^ ^]^ Nevertheless, the application of these materials presents a series of challenges. The hurdles associated with stability and reactivity are significant when it comes to the manipulation of chalcogens across diverse applications. The pronounced reactivity of oxygen is challenging in certain environments, whereas the polymorphic characteristics of sulfur influence its stability and properties. The acquisition of pure chalcogen materials is problematic, attributable to their intrinsic reactivity. This complexity renders the synthesis of these materials in higher purity and the specific forms required for various applications both difficult and costly.^[^ ^245^ ^]^ The intricacies of environmental sensitivity introduce an additional dimension, as chalcogens exhibit varying responses to fluctuations in temperature, pressure, and interactions with other elements, thereby affecting their performances and stabilities in environmental degradation applications.^[^ ^246^ ^]^ Concerns regarding compatibility emerge when a chalcogen is engaged in unfavorable interactions or demonstrates restricted compatibility with other materials or components, thereby influencing overall performance and reliability.

Chalcogenide‐based materials exhibit enhanced potential for organic pollutant removal; however, the bottleneck regarding capacity, efficiency, and cyclic stability hinders their practical application. Nonetheless, the adverse effects observed at elevated doses, coupled with the challenges in managing interactions within the systems, present significant safety and performance concerns. Therefore, the necessity to enhance the development of chalcogenide‐based photocatalysts/adsorbents for commercial applications, while maintaining quality, consistency, and cost‐effectiveness, presents a considerable challenge. Confronting these challenges typically requires a combination of sophisticated synthesis methods, material engineering, a comprehensive understanding of the basic properties, and the formulation of techniques to alleviate constraints while enhancing their beneficial characteristics. It also introduces several concerns that must be tackled to optimize their operational efficacy.

Tables 3, 4 present performances of chalcogenide materials, illustrating that fundamental properties and various parameters significantly influence their activities. A multitude of essential characteristics, including structural stability, compositional stability, band‐edge alignment, photosensitivity, interfacial contacts, recombination resistance, and anti‐photocorrosion, are imperative for the photocatalytic efficacy of these materials. This further underscores the significance of shell thickness, core diameter, and optical tunability in enhancing photocatalytic efficiency. The intricate interactions of elements that influence the overall efficacy of chalcogenide photocatalysts in the photodegradation process should be studied with optimum clarity. Essential insights highlighting the importance of the core/shell architecture in improving photocatalytic efficacy, emphasizing that structural integrity, band‐edge alignment, and photosensitivity are vital for optimal light absorption and charge separation. The minimization of charge recombination losses and the enhancement of catalyst durability hinge upon the critical factors of recombination resistance, interfacial contacts, and anti‐photocorrosion. In addition, the ability to meticulously adjust shell thickness, optical characteristics, and core diameter is crucial for enhancing the charge transfer efficiency and light absorption.

Furthermore, the discrepancies in the operational parameters of various wastewater treatment techniques render any comparative analysis between them unfeasible. Ultimately, considerations such as energy consumption, the degree of mineralization, leaching, and the toxicity of the by‐products associated with each technique may provide valuable insights for determining the most suitable approach for large‐scale implementation.

While research has provided the fundamental properties, fabrication processes, and structural characteristics of chalcogenide‐based photocatalysts, it falls short of addressing the specific concerns relating to performance metrics. Consequently, additional investigation and examination are essential to achieve a more profound understanding of the interconnections among those components and their influence on the overall photocatalytic performance. It functions as an insightful foundation for grasping the essential principles underlying the design and optimization of chalcogenide‐based materials. Therefore, investigating the practicality of chalcogenide adsorbents/photocatalysts for removing contaminants by evaluating the performance metrics is of significant interest. As discussed in Section 4, the evaluations of adsorption capacity and photocatalytic efficiency in assessing the performance of chalcogenide materials are constrained, as several critical factors that govern the process are often overlooked. For example, the balanced concentrations of the adsorbates (pollutants) frequently influence the maximum adsorption capacity. Increased adsorption uptake can be observed when the adsorbents are subjected to high adsorbate concentrations. This significantly limits the applicability of adsorption capacity for the direct evaluation of the actual efficacy of adsorbents.

Based on the above, it is essential to integrate components like the partition coefficient to evaluate the sorption performance metrics of sorbents across diverse conditions in practical applications. Under ambient conditions, a low sorption capacity may be observed for numerous sorbents that have been deemed effective according to their maximum adsorption capacity measurements. Employing the partition coefficient technique to assess performance metrics could yield a more refined evaluation of the adsorption capacities of chalcogenide materials.^[^ ^247^, ^248^ ^]^ Consequently, the performance of the sorbents could be predominantly attributed to the partition coefficient, which also serves as an effective benchmark for evaluating the efficacy of various sorbent materials.^[^ ^248^, ^249^, ^250^, ^251^ ^]^ The relationships between the maximum sorption capacities and the concentrations at equilibrium under the liquid–solid systems during the adsorption processes would result in partition coefficients.^[^ ^250^ ^]^

Addressing the toxicity associated with chalcogenide materials, which encompass elements from group 16 such as sulfur, selenium, and tellurium, poses a range of intricate issues in wastewater treatment, especially environmental safety concerns in relation to the toxicity associated with selenium.^[^ ^252^, ^253^ ^]^ The chalcogenide materials, including CdS, PbSe, and HgTe, are the subject of extensive research for their applications in photocatalysis and water treatment, owing to their distinctive optoelectronic characteristics. Nonetheless, the potential for toxicity and the persistence of these substances in the environment present significant challenges. In order to address potential ecological consequences, it is imperative to implement appropriate protocols for handling, disposal, and recycling.

Moreover, the incorporation of chalcogenides can significantly affect the long‐term stability and durability of water remediation systems. Maintaining consistent efficiency across prolonged durations and varying environmental conditions is essential for their effective utilization. The introduction of chalcogenides also brings forth compatibility and interface challenges with the existing materials utilized in water and wastewater remediation. Mitigating degradation or chemical interactions at these interfaces presents a considerable concern. Ultimately, the establishment of dependable and reproducible fabrication methodologies is of paramount importance for chalcogenide‐based materials. This includes techniques for deposition, the growth of crystals, and the assembly of devices, all aimed at achieving superior performance and consistency across extensive surfaces. Confronting these challenges requires a concerted collaboration among researchers, engineers, and industry stakeholders. Their collective knowledge can facilitate the incorporation of chalcogenides into non‐noble materials and biomass‐derived waste materials while considering factors such as economic efficiency, scalability, ecological consequences, and performance reliability.

In light of increasing practicality, chalcogenide materials have demonstrated considerable potential for the removal of a wide range of organic pollutants. However, optimization problems associated with these materials are critical for various applications, guaranteeing their enduring stability and increasing production capacity. Research data indicated that although experimental efficiencies are robust, theoretical models propose even greater potential, highlighting a disparity that warrants attention. To maximize the potential of chalcogenides, forthcoming investigations should concentrate on enhancing material efficacy, durability, and scalability.

The photocatalyst process presents numerous challenges, particularly concerning the lifespan of photocatalysis, which often poses significant barriers to practical applications, aggravated by issues related to chemical and hydrothermal stability. For example, although various prevalent semiconductors like CdS and Si exhibit commendable light‐harvesting capabilities, their photochemical stabilities are inadequate for what is necessary for them to serve as dependable photocatalysts.^[^ ^254^ ^]^ Moreover, the processes of leaching, degradation, agglomeration, and aggregation could adversely affect the optical properties and photocatalytic capabilities of the active components. In addition, a significant drawback related to photocatalysis pertains to the poisoned deactivation of photocatalysts. Consequently, significant obstacles in the investigation and application of photocatalysis include the regulation of light absorption and excitation to produce charge carriers, the suppression of electron–hole recombination, the enhancement of intrinsic activity, the delay of poisoning deactivation, and the facilitation of molecular diffusion, desorption, and adsorption.^[^ ^255^ ^]^

**Figure 21:**
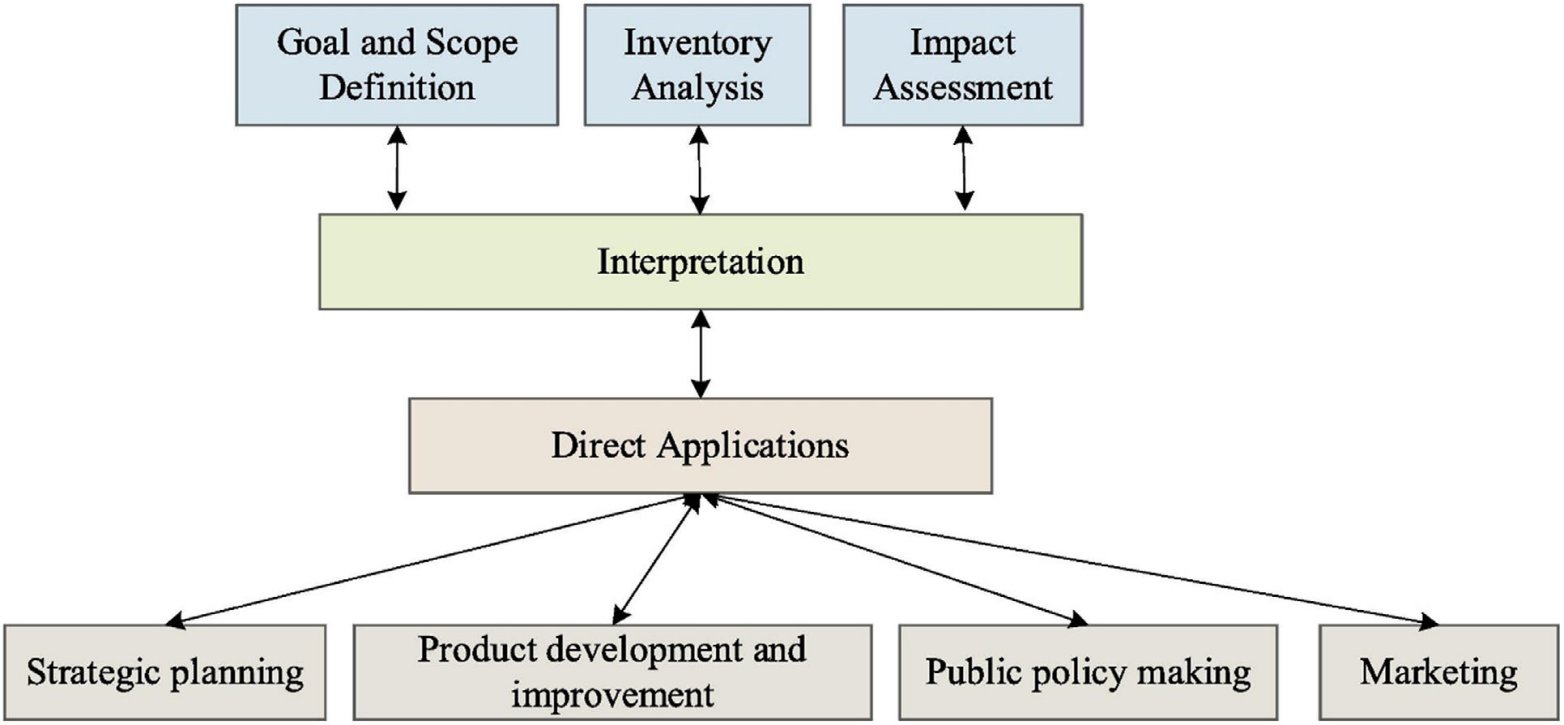
The framework for LCA.

Life cycle assessment (LCA) is currently absent in the literature surveyed on the applications of chalcogenide materials for organic pollutants remediation. However, we recommend that future studies should include the LCA, which is expected to help in translating the wealth of scientific information into actionable guidance for both researchers and practitioners in the water treatment field. LCA represents a scientifically grounded approach comprehensive approach to analyzing the environmental consequences linked to every phase of a product's life, encompassing the extraction of raw materials, production, utilization, and eventual disposal. It recognizes critical environmental areas and enhances the product's framework without transferring the burden to other domains. The process involves the meticulous compilation of a detailed inventory of inputs, including fuel, chemicals, raw materials, and water, alongside the quantification of outputs such as emissions, products, and by‐products. In the context of chalcogenide‐based treatment systems, which incorporate compounds like sulfur, selenium, or tellurium, LCA would offer valuable insights into their environmental impacts and highlight potential avenues for enhancement. This assessment aims to evaluate the potential environmental impacts derived from the entire life cycle of a product.^[^ ^256^, ^257^, ^258^ ^]^ The systematic approach for executing a LCA is illustrated in **Figure** **21**, which encompasses the following stages: i) defining the goal and scope, ii) compiling the life cycle inventory (LCI), iii) evaluating the life cycle impacts associated (LCIA) in the entire process, and iv) interpreting the results.

Noting that the objective of LCA delineates the prospective application and target audience of the research, the scope establishes the parameters of the system under examination, and the functional unit acts as the benchmark for the analysis. The LCIA creates a linkage between the process or the product and the associated potential environmental consequences. The outcomes would serve as a foundational reference for decision‐making in alignment with the established objectives and parameters. This serves as a means of comparison to assess the various environmental trade‐offs associated with products that offer similar functionalities yet differ in their forms or processes.^[^ ^257^, ^259^, ^260^ ^]^

## 7 Conclusion

Chalcogenides are a hot subject in the nanotechnology field and have a wider range of semiconducting, electrochemical, optoelectronic, and catalytic applications. This is due to their adaptable chemical composition, electronic, semiconducting, and optical properties that can be fine‐tuned. Technological advancements in chemical synthesis have fueled this development by making a variety of chalcogenide compounds in conveniently processable sizes and forms. Though these materials with an extensive array of morphologies and compositions have been developed, most of them have thick structures with very little porosity and inaccessible interfacial regions, which severely restricts their usage in several important technical fields.

The review presents the state‐of‐the‐art synthesis of chalcogenide compounds and the corresponding applications in removing organic pollutants from water/wastewater. So far, the “hard‐template method” and “oxide‐to‐sulfide conversion” approach have been effective in synthesizing ordered chalcogenide materials. Nevertheless, the hard‐templating process hinders the capacity to fine‐tune the pore architectures of chalcogenides and their broad adoption due to its considerable constraints, such as its lack of synthesis flexibility, higher cost, and larger time‐consuming issues. Instead, well‐defined uniform pores could be formed utilizing the easier soft‐template processes that use cluster‐ion or molecular precursor, which allow a higher degree of influence over the shape and pore size. Employing specified nanomaterial(s) as building block, soft‐template‐assisted synthetic method of chalcogenide with nanocrystalline colloidal material allows for precise control of the local structure, hence avoiding the problems of unregulated crystal development and the consequent loss of porosity.

Even though a lot of success in fabricating chalcogenides and their possible applications for water/wastewater treatment has been reported, there exists a number of innovations in synthetic protocols that can create nanostructures with unique shapes and properties. In a sustainable society, these innovations might open doors to useful uses. One very difficult task for synthetic chemists is to assemble connected chalcogenide nanocrystals into well‐ordered mesoporous structures. The main issue is the intricate nature of the process, which necessitates the exact manipulation of the nanoscale spatial configuration of nano building fragments. Because they depend on the weak intramolecular relationships among colloidal nanoparticles, conventional approaches like supramolecular self‐assembly do not provide full control over the spatial arrangement and so are not entirely successful. These materials are ideal because they can incorporate complementary functionalities like nano porosity and distinct photocatalytic and electrical characteristics of individual nanoparticles into one integrated structure. In addition, mesoscopically ordered arrays of chalcogenide nanoparticle(s) may exhibit additional collective functions not seen in the main materials, such as modified electrochemical properties and new kinds of complementary host–guest interaction. Investigating the unbound chemistry and potential of chalcogenides reveals diverse self‐assemblage principles involving structural components, variable structural and functional regulation, and dynamic arrays of novel functions.

There is a need to optimize catalysts derived from chalcogenide materials. This is because excessive chalcogen results in agglomeration, which covers the active sites and lowers conductivity/activity, thereby impacting catalytic performance. It should be noted that they attract inhibitors and increase the negative charges.

## Conflict of Interest

The authors declare no conflict of interest.
